# Synthesis and Characterization of a Crystalline Imine-Based Covalent Organic Framework with Triazine Node and Biphenyl Linker and Its Fluorinated Derivate for CO_2_/CH_4_ Separation

**DOI:** 10.3390/ma15082807

**Published:** 2022-04-11

**Authors:** Stefanie Bügel, Malte Hähnel, Tom Kunde, Nader de Sousa Amadeu, Yangyang Sun, Alex Spieß, Thi Hai Yen Beglau, Bernd M. Schmidt, Christoph Janiak

**Affiliations:** 1 Institut für Anorganische Chemie und Strukturchemie, Heinrich-Heine-Universität Düsseldorf, 40204 Düsseldorf, Germany; stefanie.buegel@hhu.de (S.B.); mahae107@hhu.de (M.H.); yasun100@hhu.de (Y.S.); alex.spiess@hhu.de (A.S.); beglau@hhu.de (T.H.Y.B.); 2 Institut für Organische Chemie und Makromolekulare Chemie, Heinrich-Heine-Universität Düsseldorf, 40204 Düsseldorf, Germany; kunde@hhu.de; 3 Bundesanstalt für Materialforschung und -Prüfung, Fachbereich 6.3 (Strukturanalytik), 12489 Berlin, Germany; nader.amadeu@bam.de

**Keywords:** covalent organic framework (COF), imine-COF, fluorinated COF, mixed-matrix membrane (MMM), CO_2_/CH_4_ separation

## Abstract

A catalyst-free Schiff base reaction was applied to synthesize two imine-linked covalent organic frameworks (COFs). The condensation reaction of 1,3,5-tris-(4-aminophenyl)triazine (TAPT) with 4,4′-biphenyldicarboxaldehyde led to the structure of HHU-COF-1 (HHU = Heinrich-Heine University). The fluorinated analog HHU-COF-2 was obtained with 2,2′,3,3′,5,5′,6,6′-octafluoro-4,4′-biphenyldicarboxaldehyde. Solid-state NMR, infrared spectroscopy, X-ray photoelectron spectroscopy, and elemental analysis confirmed the successful formation of the two network structures. The crystalline materials are characterized by high Brunauer–Emmett–Teller surface areas of 2352 m^2^/g for HHU-COF-1 and 1356 m^2^/g for HHU-COF-2. The products of a larger-scale synthesis were applied to prepare mixed-matrix membranes (MMMs) with the polymer Matrimid. CO_2_/CH_4_ permeation tests revealed a moderate increase in CO_2_ permeability at constant selectivity for HHU-COF-1 as a dispersed phase, whereas application of the fluorinated COF led to a CO_2_/CH_4_ selectivity increase from 42 for the pure Matrimid membrane to 51 for 8 wt% of HHU-COF-2 and a permeability increase from 6.8 to 13.0 Barrer for the 24 wt% MMM.

## 1. Introduction

Covalent organic frameworks (COFs) are porous crystalline materials built entirely by covalent bonds between light elements. Since the first COFs, namely COF-1 and COF-5, were synthesized in 2005 [1], research on COFs has been rapidly developing. The opportunity to change, for example, the linkers or the linkage leads to a broad variety of two-dimensional (2D) or three-dimensional (3D) structures [2]. The associated tunable material characteristics open up the possibility of applications in various areas such as gas storage [3], molecular separation [4,5,6], catalysis [7], sensing [8], energy storage [9], and optoelectronics [10].

COFs synthesized via a condensation reaction of an aldehyde and an amine represent the class of imine-COFs. The dynamic formation process based on Schiff base chemistry results in crystalline and porous materials with high chemical and thermal stability [11]. In 2009, the first imine-COF (COF-300) was synthesized from tetra-(4-anilyl)methane and terephthalaldehyde (TA), resulting in a crystalline 3D COF with diamond topology. COF-300 exhibited permanent porosity with a Brunauer–Emmett–Teller (BET) surface area of 1360 m^2^/g and thermal stability up to 490 °C [12]. Two years later, the first 2D imine-COF was reported by Ding et al. The condensation of 1,3,5-triformylbenzene and 1,4-phenylenediamine yielded the layered COF-LZU1, with hexagonal channels and narrow pores with a distribution around 1.2 nm [13]. Based on this hexagonal 2D structure, more nitrogen-rich COFs were synthesized by using 1,3,5-tris-(4-aminophenyl)triazine (TAPT) and TA in a Schiff base reaction. Gomes et al. applied these educts to synthesize the porous polymer TRITER-1 (Scheme S1) with a BET surface area of 716 m^2^/g [14]. In a solvothermal catalyst-free (SCF) synthesis, Liao et al. successfully produced a fluorinated analog by substituting the educt TAPT with 2,3,5,6-tetrafluoroterephthalaldehyde (TFTA). The resulting SCF-FCOF-1 (Scheme S1) showed a suitable crystallinity and a large BET surface area of 2056 m^2^/g [15].

Nitrogen-containing covalent triazine frameworks (CTFs) [16,17,18,19] and fluorine-containing porous materials (metal-organic frameworks; MOFs [20,21] and COFs, including CTFs [22,23,24]) are sought for their potentially high CO_2_ sorption and separation properties. The incorporation of these porous materials as fillers into polymer matrices as a continuous phase leads to the formation of mixed-matrix membranes (MMMs), and the composites are promising for CO_2_/CH_4_ separation as an application [25,26,27,28]. While examples of MOF- and COF-based MMMs are frequent, there are fewer cases of CTF-based MMMs [29,30]. With the microporous azine-linked COF material ACOF-1 as a filler in Matrimid, the gas separation performance of the resulting MMM was tested with an equimolar mixture of CO_2_/CH_4_ at 308 K. With 16 wt% filler, the CO_2_ permeability was increased from 6.8 Barrer for the pure Matrimid membrane to 15.3 Barrer, with no loss of CO_2_/CH_4_ selectivity [31]. A total of 5 wt% of the fluorinated CTF, FCTF-1, embedded in a PIM-1 matrix led to an improvement in CO_2_ permeability from 5800 to 9400 Barrer. In this case, it was also possible to improve the CO_2_/CH_4_ selectivity from 11.5 to 14.8 [32]. These examples show that higher membrane efficiency can be achieved by incorporating fillers into membranes. Important prerequisites are suitable compatibility between filler and polymer and uniform distribution of the filler without sedimentation.

**Scheme 1:**
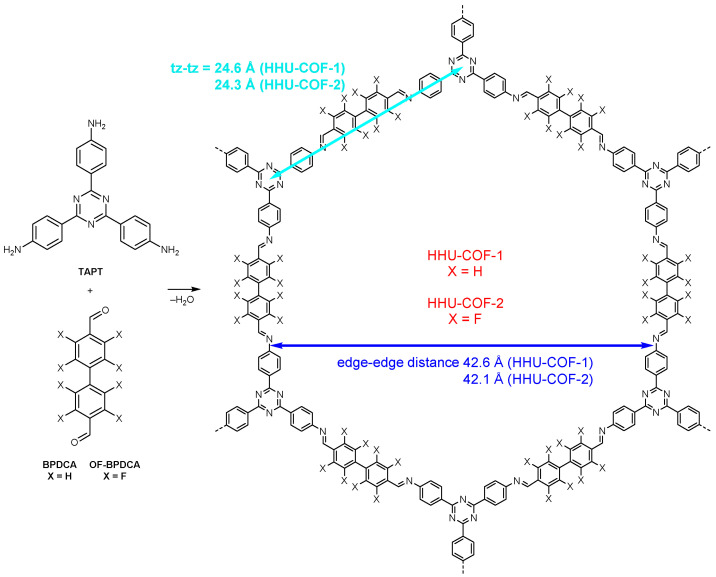
Schematic formation of HHU-COF-1 and HHU-COF-2 from TAPT and BPDCA or OF-BPDCA, respectively, indicating the idealized hexagonal ring and network in the HHU-COF products (HHU = Heinrich-Heine University). The given triazine-centroid triazine-centroid (tz-tz) distance along the edges and the edge-edge distances were determined from the most intense (100) reflexes in the powder X-ray diffractograms, assuming a hexagonal unit cell for the honeycomb layer (cf. Figure S16). The given tz-tz distance along the edge is half the tz-tz distance across the ring.

In this work, we present two large-pore imine-COFs with a triazine node, akin to CTFs, which were synthesized via the catalyst-free Schiff base reaction at 120 °C to avoid any nitrogen and fluorine loss. The standard ionothermal CTF synthesis not only leads to a large loss of nitrogen but also to defluorination during the reaction at >400 °C with high equivalents of ZnCl_2_ [23,24]. Here, the triazine-based node TAPT and the linkers 4,4′-biphenyldicarboxaldehyde (BPDCA) and 2,2′,3,3′,5,5′,6,6′-octafluoro-4,4′-biphenyldicarboxaldehyde (OF-BPDCA) were reacted to give the crystalline COFs HHU-COF-1 and HHU-COF-2 (Scheme 1). OF-BPDCA features an inverse electron density distribution due to its fluorine substituents compared to BPDCA and exhibits a larger dihedral angle of 36° between the two aromatic rings (BPDCA 18°). OF-BPDCA has been used in the assembly of helical channels in supramolecular organic frameworks but not as a linker in COFs [33]. In addition, the combination of BPDCA with TAPT to form a COF is novel to the best of our knowledge [11]. Further, the nitrogen-rich structures, which are favorable for CO_2_ adsorption due to quadrupole-dipole interactions [34], were applied as filler materials to form MMMs for CO_2_/CH_4_ mixed-gas separation. The MMM matrix was Matrimid, a glassy polymer with intrinsically high CO_2_/CH_4_ selectivity [35,36].

## 2. Materials and Methods

### 2.1. Materials

For COFs: 4,4′-Biphenyldicarboxaldehyde (BPDCA; 97%) was obtained from ACROS Organics and 1,3,5-tris-(4-aminophenyl)triazine (TAPT; >98%) from TCI. 2,2′,3,3′,5,5′,6,6′-octafluoro-4,4′-biphenyldicarboxaldehyde (OF-BPDCA) was synthesized as described in Section S1 of the Supplementary Materials. The COF syntheses were carried out in analogy to the synthesis of SCF-FCOF-1 [15]. All solvents were purchased from commercial suppliers with a minimum purity of 99.8%.

For membranes: Matrimid^®^ 5218 (BTDA/DAPI) was provided by Huntsman Advanced Materials, and dichloromethane (DCM; 99.99%) was obtained from Fisher Scientific (Hampton, NH, USA).

Gases: CO_2_ (grade 4.5), CH_4_ (grade 4.5), and He (grade 5.0) were received from Air Liquide.

### 2.2. Synthesis of HHU-COF-1

A total of 84.1 mg (0.400 mmol) of BPDCA, 94.5 mg (0.267 mmol) of TAPT, and 1 mL of the solvent mixture of 1,4-dioxane and mesitylene (1:1, *v*/*v*) were placed in a glass ampoule, followed by an ultra-sonification treatment for 15 min in order to ensure sufficient mixing of the educts. The mixture was degassed by applying three freeze-pump-thaw cycles, and the ampoule was flame sealed under vacuum. After heating at 120 °C for three days, the crude product was washed with THF, followed by Soxhlet extraction for 24 h each in THF and in ethanol to remove unreacted monomers. Drying was performed with supercritical CO_2_ (yield: 147 mg; 89.5%).

### 2.3. Synthesis of HHU-COF-1 (Larger Scale)

A total of 252.3 mg (1.200 mmol) of BPDCA, 283.5 mg (0.800 mmol) of TAPT, and 4 mL of the solvent mixture of 1,4-dioxane and mesitylene (1:1, *v*/*v*) were placed in a glass ampoule, followed by an ultra-sonification treatment for 30 min and the procedures described in 2.2 (yield: 433 mg; 87.9%).

### 2.4. Synthesis of HHU-COF-2

A total of 70.8 mg (0.200 mmol) of OF-BPDCA, 47.2 mg (0.133 mmol) of TAPT, and 3 mL of the solvent mixture of 1,4-dioxane and mesitylene (1:1, *v*/*v*) were placed in a glass ampoule, followed by an ultra-sonification treatment for 30 min and the procedures described in 2.2 (yield: 93 mg; 84.3%).

### 2.5. Synthesis of HHU-COF-2 (Larger Scale)

A total of 354.3 mg (1.000 mmol) OF-BPDCA, 236.4 mg (0.667 mmol) TAPT, and 6 mL of the solvent mixture of 1,4-dioxane and mesitylene (1:1, *v*/*v*) were placed in a glass ampoule, followed by an ultra-sonification treatment for 50 min and the procedures described in 2.2 (yield: 485 mg; 87.4%).

### 2.6. Preparation of HHU-COF-1/Matrimid and HHU-COF-2/Matrimid MMMs

To ensure that all MMMs are prepared from the same batch, the COF materials obtained from the larger-scale syntheses were applied as filler materials in the MMMs. Characterization of these COFs can be found in Section S3, Supplementary Materials. The HHU-COF-1/Matrimid and HHU-COF-2/Matrimid MMMs were prepared by solution casting with filler loadings of 8, 16, and 24 wt% (Figures S23 and S24). The filler loading was calculated according to Equation (1):

(1) $$Filler\;loading\;\left[wt\%\right]=\frac{m_{filler}}{m_{polymer}+m_{filler}}\times 100\;\%$$

The 8 wt% membranes were prepared as follows: 250 mg of Matrimid (stored at 80 °C) were dissolved in 3 mL of DCM. A total of 22 mg (48 mg for 16 wt% and 79 mg for 24 wt%) of COF were dispersed in 4 mL of DCM. Both polymer and COF were stirred for 24 h. The COF dispersion was ultrasonicated using a Microtip 630-0419 (VCX 750 Sonics) three times for 15 min (amplitude of 20%) with 30 min stirring between the ultra-sonification steps. Afterward, 0.29 mL (0.62 mL for 16 wt% and 1.01 mL for 24 wt%) of the Matrimid solution was added to the COF dispersion, followed by stirring for another 24 h. The same ultra-sonification procedure was repeated, and the remaining Matrimid solution was added to the COF dispersion. After stirring for two hours, the final dispersion was casted into a metal ring placed on a flat and even glass surface. To ensure slow evaporation of DCM, an inverted funnel covered with a paper tissue was placed above the MMM until the DCM was evaporated. The membrane was cut out of the metal ring with a scalpel. The membranes were dried at 150 °C in a vacuum oven (20 mbar) overnight.

### 2.7. Instrumentation and Characterization Methods

Supercritical CO_2_ drying was conducted with an EM CPD300 (Leica, Wetzlar, Germany). Ethanol was exchanged for CO_2_ with 50 cycles for each product. Solid-state NMR experiments were performed with an AVANCE 600 spectrometer (Bruker, Billerica, MA, USA) under a static field of 14 T. The samples were packed into 2.5 mm zirconia rotors with Vespel top and bottom plugs and spun at 35 kHz under the magic angle (MAS) in a double resonant wide-bore probe at room temperature. Typical acquisition parameters for the ^13^C MAS NMR spectra were a repetition period (d1) equal to or higher than 12 s; 9 ms contact time (cross-polarization), and 1–2 µs for the 90° pulses. During a typical acquisition time of about 10 ms, ^1^H was decoupled using the spinal64 sequence. Very long experiments with accumulations between 16 and 64 k scans were performed in order to overcome the low sensitivity observed for those samples. For ^19^F solid-state NMR experiments, the sample was spun in a 2.5 mm zirconia rotor with Vespel top and bottom plugs at 35 kHz (MAS). The EASY pulse program [37] was applied, in which two 90° pulses were irradiated with a little delay in between. By subtracting the FID after each of those pulses, the background signal was canceled. Subsequent processing involved Fourier transformation, phase adjustment, and baseline correction (Bernstein polynomials, fourth-order or higher). Attenuated total reflection infrared spectroscopy (ATR-IR, platinum ATR-QL, diamond) spectra were obtained on a Tensor 37 (Bruker). X-ray photoelectron spectroscopy (XPS) measurements were performed on a VersaProbe II (Ulvac-Phi) with a monochromatic Al X-ray source (1486.6 eV). The C1s signal at 284.8 eV was taken as the reference for the binding energy scale. Analysis of the spectra was carried out with the program CasaXPS. C, H, N-analysis was carried out on a Vario MICRO cube from Elementar Analysentechnik. Scanning electron microscopy (SEM) images were recorded with a JSM-6510LV (Jeol) with a LaB_6_ cathode (20 keV). The membrane cross-sections were obtained by freeze-fracturing with liquid nitrogen and coated with gold by a JFC 1200 (Jeol) sputter coater for imaging. Energy-dispersive X-ray spectroscopy (EDX) was carried out using an Xflash silicon drift detector (Bruker). Thermogravimetric analysis (TGA) was performed under a nitrogen atmosphere with a TG 209 F3 Tarsus (Netzsch, Selb, Germany) in a range from 25 to 1000 °C with a heating rate of 5 K/min.

An Autosorb-6 (Quantachrome, Boynton Beach, FL, USA) was used to perform the nitrogen sorption measurements. Prior to the measurements, all samples were activated by degassing under vacuum at 120 °C for 8 h. The Brunauer–Emmett–Teller (BET) surface area was calculated from the range of 0.05 to 0.3 p/p_0_ of the nitrogen adsorption isotherms. CO_2_ and CH_4_ sorption isotherms were obtained with an Autosorb iQ MP (Quantachrome). The samples were activated in vacuo (5 × 10^–3^ mbar) at 120 °C for 8 h. In order to calculate the ideal adsorbed solution theory (IAST) selectivity for the gas pair CO_2_/CH_4,_ the sorption isotherms of HHU-COF-1 were fitted with the Langmuir isotherm (LAI) model by applying Equation (2):

(2) $$q_{eq}=q_{max}\times \frac{K\times p}{1+K\times p}$$

The isotherms of HHU-COF-2 were fitted with the Toth isotherm model (Equation (3)),

(3) $$q_{eq}=q_{max}\times \frac{K\times p}{{\left(1+K\times p\right)}^{t}{)}^{1/t}}$$

where q and K refer to the loading in mmol/g and the affinity constant, respectively. The heterogeneity exponent is expressed as t with the unit 1/bar. After isotherm fitting, the final IAST selectivity was calculated by Equation (4), where $x_{i}$ is the absorbed gas amount in mmol/g and $y_{i}$ is the mole fraction.

(4) $$S=\frac{x_{1}/y_{1}}{x_{2}/y_{2}}$$

Powder X-ray diffraction (PXRD) patterns were obtained from a Miniflex 600 (Rigaku, Tokyo, Japan) using Cu Kα1 radiation with λ = 1.5406 Å (40 kV, 15 mA, 600 W) and a flat silicon low background sample holder in the range of 2° < 2θ < 50°. For determination of the (skeletal) density, a He-pycnometer AccuPyc 1330 (Micromeritics) was used. The density applied for calculations was obtained by including the total pore volume from N_2_-sorption as shown in Equation (5):

(5) $$\rho_{COF}=\frac{m_{COF}}{V_{He}+V_{pores}}$$

The tensile strength of the pure Matrimid membrane and MMMs was measured using a TAXT.plus texture analyzer (Stable Micro Systems). The force (N) was divided by the product of the thickness and width of the membrane to obtain the tensile strength to break the membrane in MPa.

CO_2_/CH_4_ mixed-gas separation measurements were carried out with an OSMO inspector (Convergence Industry B.V.) connected to an Agilent 490 Micro GC (Agilent Technologies, Santa Clara, CA, USA) with a fused silica column PoraPLOT Q and a thermal conductivity detector. A diagram of the experimental setup is shown in Figure S25. The membranes were placed in the permeation module and fixed with a Viton O-ring with an inner diameter of 3.6 cm (area: 11.3 cm^2^) inside the permeation module. A CO_2_/CH_4_ feed gas volume ratio of 1:1 was applied, and helium was used as the sweep gas. The transmembrane pressure and the temperature were set to 3 bar and 25 °C, respectively. All measurements were carried out every 30 min until the equilibrium state was reached. The permeability P is expressed in the unit Barrer (1 Barrer = 10^–10^ cm^3^(STP) cm cm^–2^ s^−1^ cmHg^−1^) and was calculated according to Equation (6):

(6) $$P=\frac{x_{A}\times Q_{He}\times d\;}{x_{He\;}\times A\times \left(p_{2}\times x_{A}^{f}-p_{1}\times x_{A}\right)}$$

$x_{A}$ describes the molar fraction of the gas A, $Q_{He}$ is the volumetric flow rate of the sweep gas helium, d is the thickness of the membrane (measured at 10 different points) and $x_{He}$ is the molar fraction of the sweep gas. *A* stands for the area of the membrane (cm^2^), $p_{2}$ for the feed pressure (cmHg), $x_{A}^{f}$ for the molar fraction of the gas A in the feed, and $p_{1}$ for the permeate pressure (cmHg). The molar fractions (*x*) on the permeate and the feed side were used to calculate the selectivity of the two gases (*A*, *B*), according to Equation (7):

(7) $$\alpha_{A,B}=\frac{{\left(x_{A}/x_{B}\right)}_{permeate\;side}}{{\left(x_{A}/x_{B}\right)}_{feed\;side}}$$

## 3. Results and Discussion

### 3.1. Characterization of HHU-COF-1 and HHU-COF-2

**Figure 1:**
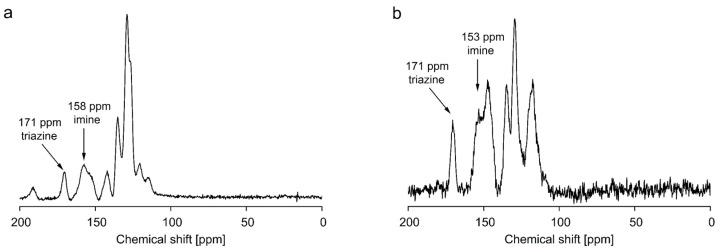
^13^C CP MAS NMR spectra of (**a**) HHU-COF-1 and (**b**) HHU-COF-2. Spectra were obtained at 150 MHz, cross-polarization with decoupling spinal and 35 kHz spinning.

**Figure 2:**
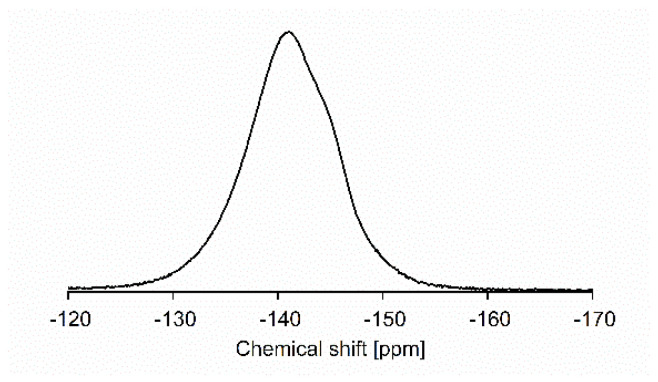
^19^F CP MAS NMR spectra of HHU-COF-2.

The Schiff base reaction of 1,3,5-tris-(4-aminophenyl)triazine (TAPT) with either 4,4′-biphenyldicarboxaldehyde (BPDCA) or the corresponding octafluoro derivative OF-BPDCA yielded the two COFs HHU-COF-1 and HHU-COF-2 as yellow and orange powder (Scheme 1, Figure S17). A three-day synthesis was carried out in ampoules to allow for a slow, reversible reaction leading to crystalline products. Both COFs were reproducibly synthesized on the scale of 0.13 to 0.8 mmol of TAPT in a 1:1.5 ratio with (OF-)BPDCA under the same reaction conditions (cf. Supplementary Materials). Yields ranging from 84% to 90% could be reached. ^13^C CP MAS NMR spectra confirmed the formation of the imine bond at 158 ppm for HHU-COF-1 and 153 ppm for HHU-COF-2 (Figure 1). These values agree well with the related TRITER-1 (158 ppm) [14] and SCF-FCOF-1 (152 ppm) [15] for their carbon atoms of the imine bond. Both COFs exhibited a peak at 171 ppm, referring to the carbon atoms of the triazine ring again identical to TRITER-1 [14]. The signals in the range from 120 to 150 ppm can be assigned to the carbon atoms in the benzene rings. For HHU-COF-1, the small peak at 191 ppm can be attributed to some unreacted aldehyde [38]. Because of the extensive purification with Soxhlet extraction, we assume that the aldehyde signal did not originate from unreacted monomers and is instead caused by partially incomplete network formation, that is, defect sites. In the ^19^F CP MAS NMR spectrum of HHU-COF-2 (Figure 2), the two peaks expected from solution NMR of OF-BPDCA at −136 and −144 ppm (Figure S3) can only be seen as one peak at −141 ppm due to the line broadening in solid-state NMR.

**Figure 3:**
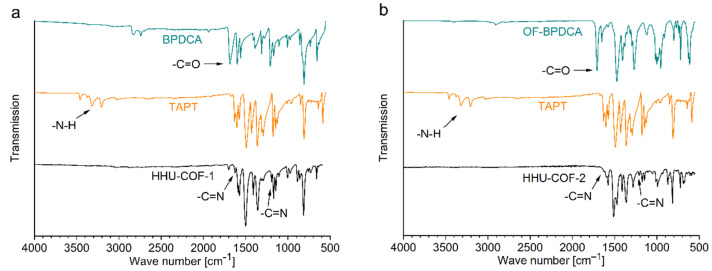
IR-spectra of (**a**) HHU-COF-1 and (**b**) HHU-COF-2 in comparison with the spectra of the educts.

FT-IR spectra (Figure 3) showed weak bands at 1626 and 1193 cm^−1^ for HHU-COF-1 and at 1624 and 1211 cm^−1^ for HHU-COF-2, which could be assigned to the imine bond [12]. N–H stretching bands from the amino groups in TAPT, which typically appear between 3500 and 3300 cm^−1^, were no longer visible in the spectra of the two COFs, proving the conversion of the amine groups and, therefore, the consumption of the linker molecule TAPT. The C=O stretching band of BPDCA at 1686 cm^−1^ can still be detected as a small band in the spectrum of HHU-COF-1 (at 1700 cm^−1^), indicating residual aldehyde, as was already seen in the ^13^C NMR spectrum (Figure 1a). The absence of the C=O stretching band of OF-BPDCA at 1708 cm^−1^ in the respective COF indicates a complete conversion of the aldehyde groups of the educt in HHU-COF-2.

The XPS survey spectra in the range of binding energies from 0 to 1100 eV can be seen in Figure S7. In this overview, C1s peaks and N1s peaks are visible for both COFs, as well as additional peaks in the F1s and F2s region. In the high-resolution C1s XPS spectra of HHU-COF-1 (Figure S8), the peaks at 284.8 and 286.2 eV were assigned to the carbon-carbon/carbon-hydrogen bonds and the carbon-nitrogen bonds (C=N-C), respectively [39]. Fitting of the N1s region revealed a peak at 398.8 eV corresponding to the imine bond formed by the Schiff base reaction as well as the carbon-nitrogen bonds in the triazine unit [22,40]. For HHU-COF-2, the corresponding peaks in the C1s region (Figure S9) were located at 284.6 and 286.1 eV. The additional peak at 287.8 eV refers to the C-F bonds. The nitrogen-carbon bonds are evident in the N1s region, with a peak at again 398.8 eV [39]. The F1s peak was detected at 687.7 eV (Figure S10), and by integrating the peak area, a fluorine content of 19.5 at%, which corresponds to 27.3 wt% within C, N, and F, was determined for HHU-COF-2 (Table S1), which matches well with the calculation of 27.4 wt% from the ideal formula. Hydrogen cannot be detected by XPS, so the H wt% has to remain unaccounted for. Its missing share increases the wt% of C, N, and F.

The CHN elemental analysis of HHU-COF-1 and HHU-COF-2 in Table 1 was in suitable accordance with the calculated theoretical values for both COFs. In agreement with the XPS data (F wt% = 27.3 wt%), the residual content of 26.78 wt% in the CHN elemental analysis for HHU-COF-2 can be mainly attributed to the fluorine content.

**Figure 4:**
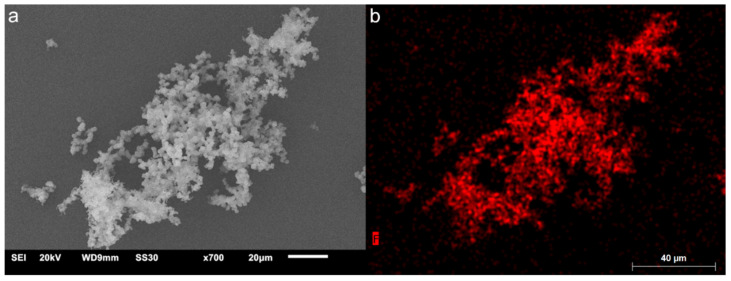
(**a**) SEM image of HHU-COF-2 and (**b**) associated fluorine elemental mapping.

A uniform distribution of fluorine in HHU-COF-2 was shown with element mapping by SEM-EDX (Figure 4). SEM images exhibited agglomerated spherical particles with an average size between 2 and 5 µm in diameter for both COFs (Figure 4, Figures S11 and S12).

**Figure 5:**
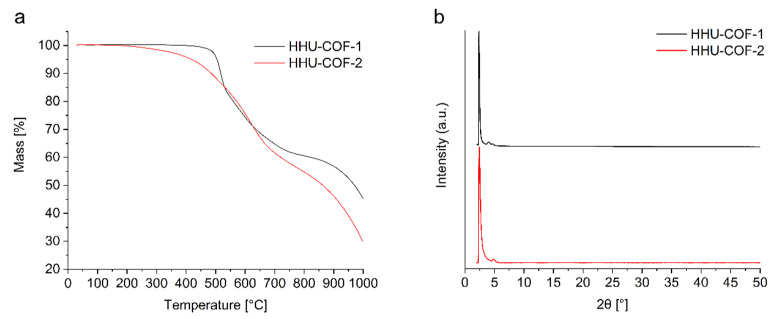
(**a**) TGA curves acquired under nitrogen atmosphere with a heating rate of 5 K/min and (**b**) PXRD pattern of HHU-COF-1 and HHU-COF-2. The first derivative of the TGA curves is given in Figure S15.

The thermal stabilities of the two COFs were investigated by TGA measurements under a nitrogen atmosphere (Figure 5a). The decomposition of HHU-COF-1 started at 450 °C with a maximum weight loss of about 40% up to 800 °C and ~55% at 1000 °C. For HHU-COF-2, gradual decomposition started at the lower temperature of ~180 °C and rather steadily continued up to 1000 °C, amounting to a weight loss of 70%. In the related TRITER-1 and SCF-FCOF-1, decomposition started at 480 and 430 °C, respectively (Figure S32).

PXRD patterns (Figure 5b) confirmed the crystalline nature of the materials. For HHU-COF-1 and HHU-COF-2, an intense diffraction peak was observed at 2.39° 2θ (d = 36.9 Å) and 2.42° 2θ (d = 36.5 Å), respectively, which corresponds to the peak at 2.93° 2θ (d = 30.2 Å) for SCF-FCOF-1 and 3.11° 2θ (d = 28.4 Å) for the (100) plane [15]. Two additional peaks for HHU-COF-1 were found at 4.03° (d = 21.9 Å) and 4.68° 2θ (d = 18.9 Å) for the (110) and (200) planes. HHU-COF-2 exhibited a smaller peak at 4.06° (d = 21.8 Å), barely detectable under the broadening of the most intense peak, and another peak at 4.85° 2θ (d = 18.2 Å). Compared to the values for SCF-FCOF-1 at 4.98° and 5.85° 2θ for the (110) and (200) planes, respectively [15], the peaks for the HHU-COFs were shifted to lower 2θ values due to the larger linker molecule. If one assumes a honeycomb (**hcb**) lattice with almost eclipsed stacked layers, the three measured peaks and corresponding (100), (110), and (200) planes can be matched to the edge-edge distance of the hexagonal rings (Scheme 1, Figure S16). Notably, a diffraction peak at about 25°–26° 2θ for the (001) plane from the interlayer π-π stacking is not seen.

**Figure 6:**
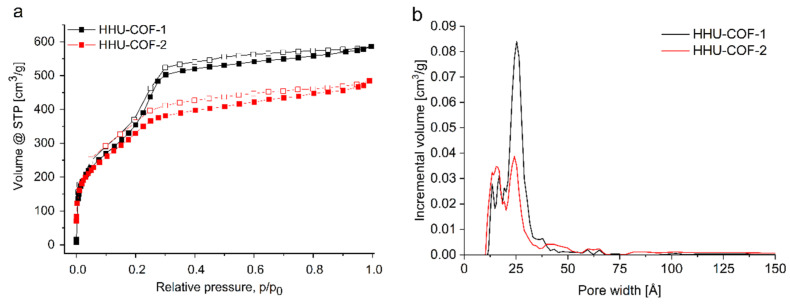
(**a**) Nitrogen sorption isotherms at 77 K and (**b**) pore size distributions of HHU-COF-1 and HHU-COF-2.

N_2_-sorption (Figure 6a) showed type IV(a) isotherms for both COFs, which turn into a type II or III isotherm at higher relative pressure. The type IV isotherm with a narrow H1 hysteresis loop reflects the micro-mesoporous nature of the COFs. The type II or III isotherm with an H4 hysteresis reflects the condensation of N_2_ in the macroporous interparticle voids from the aggregation of the COF particles [41]. HHU-COF-1 and HHU-COF-2 exhibited high BET surface areas of 2352 m^2^/g (p/p_0_ = 0.18–0.28) and 1356 m^2^/g (p/p_0_ = 0.15–0.25), respectively. The total pore volumes (at p/p_0_ = 0.97) of HHU-COF-1 and its fluorinated analog were determined as 0.78 and 0.73 cm^3^/g, respectively. We note that the trend in surface area was reversed for the TA analogs SCF-HCOF-1 (S_BET_ = 318 m^2^/g, no V_pore_ given) and SCF-FCOF-1 (S_BET_ = 1602–2056 m^2^/g, 5 h to 3 d synthesis time, V_pore_ = 1.69 cm^3^/g) [15]. However, the data for SCF-HCOF-1 cannot be taken as representative since the product was obtained in only 45% yield (96% for SCF-FCOF-1) and was noted to be of poor crystallinity, possibly due to the low activity of the TA under the solvothermal catalyst-free (SCF) synthesis conditions [15]. Under a 12 h reflux synthesis in DMF, the SCF-HCOF-1-identical TRITER-1 (cf. Scheme S1) showed a BET surface area of 716 m^2^/g [14]. In this respect, it is remarkable that BPDCA and its octafluoro-congener exhibit no differences in reactivity and yield. Concerning the high porosity of the SCF-FCOF-1 products with the triazine ring node, it is peculiar that the SCF-FCOF-2 analog with the benzene node (cf. Scheme S1) has only a BET surface area of 1245 m^2^/g and a pore volume of 0.64 cm^3^/g. We also synthesized the SCF-HCOF-1 (TRITER-1) and SCF-FCOF-1 with the smaller linkers TA and TFTA (Scheme S1, Figure S34). The synthesis and analytical results can be found in Section S5, Supplementary Materials. From our synthesis it was possible to obtain BET surface areas for SCF-HCOF-1 of 977 m^2^/g (V_pore_ = 0.64 cm^3^/g) and for SCF-FCOF-1 of 2276 m^2^/g (V_pore_ = 1.46 cm^3^/g). Although it was possible to generate SCF-HCOF-1 with a larger surface area, the reversed trend in surface area is still evident for the HHU-COFs with the larger linkers. This was also observed with the larger-scale products (Section S3, Supplementary Materials), where HHU-COF-1 (larger scale) exhibited the larger BET surface area of 2351 m^2^/g (V_pore_ = 0.69 cm^3^/g) compared to HHU-COF-2 (larger scale) with 1346 m^2^/g (V_pore_ = 0.68 cm^3^/g). A similar trend to the HHU-COFs was observed for CTFs, which were ionothermally trimerized from 4,4′-biphenyldicarbonitrile and its octafluoro derivative. The BET surface area of the fluorinated CTF was about 35% lower compared to the non-fluorinated compound [23].

Pore size distributions for HHU-COF-1 and HHU-COF-2 were calculated by applying the carbon slit-pore, non-local density functional theory (NLDFT) equilibrium model and revealed maxima at 13, 17, and 25 Å for HHU-COF-1 (Figure 6b). The maxima for HHU-COF-2 were located at 13, 16, and 24 Å. In the region of the larger ~25 Å pores, a lower incremental volume was found for HHU-COF-2, which is consistent with the presence of fluorine atoms. The diameters of the hexagonal rings in the idealized **hcb** lattice are estimated from Scheme 1 to 37–39 Å by taking into account the aryl ring radii, C-H and C-F bond lengths, and the vdW radii of the surface H and F atoms (1.2 and 1.35 Å, respectively).

In addition, CO_2_ and CH_4_ sorption studies were performed at a temperature of 273 K (Figure S13). HHU-COF-2 exhibited a higher CO_2_ uptake than the non-fluorinated HHU-COF-1, as would be expected for fluorinated materials, which are generally associated with a CO_2_-philic character [42]. HHU-COF-1 and HHU-COF-2 showed maximum CO_2_ uptakes of 1.08 and 1.74 mmol/g, respectively, which can be considered moderate compared with other imine-COFs (Table S3). The CH_4_ adsorption capacities were found to be 0.52 and 0.66 mmol/g for HHU-COF-1 and HHU-COF-2, respectively. The adsorption isotherms at 273 K were further applied to calculate the IAST selectivity for the gas pair CO_2_/CH_4_. Therefore, the isotherms of HHU-COF-1 were fitted with the Langmuir (LAI) isotherm model and the isotherms of HHU-COF-2 with the Toth model (Table S4). The CO_2_/CH_4_ selectivity (Figure S14) for a binary (50:50; *v*:*v*) mixture of the gases at 1 bar pressure was 2.1 for HHU-COF-1 and 2.6 for HHU-COF-2, respectively.

### 3.2. Characterization of MMMs

**Figure 7:**
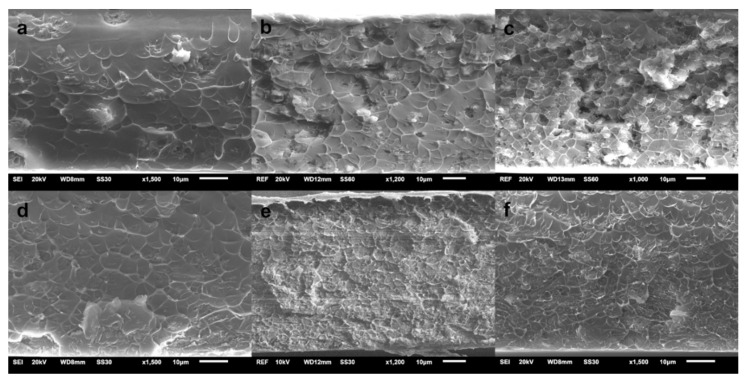
Cross-section SEM images of HHU-COF-1/Matrimid with (**a**) 8 wt%, (**b**) 16 wt%, and (**c**) 24 wt% filler and HHU-COF-2/Matrimid MMMs with (**d**) 8 wt%, (**e**) 16 wt%, and (**f**) 24 wt% filler.

In order to investigate the distribution of the dispersed phase in the Matrimid matrices, cross-section SEM images (Figure 7) were acquired. No major defects that could lead to a reduction in selectivity were observed in any of the MMMs. However, with the increasing filler content of HHU-COF-1, more minor defects became visible. This was especially the case for the 24 wt% MMM, where slight sedimentation was observed as well. For all MMMs with HHU-COF-2 as filler, a uniform distribution of the filler particles and a defect-free appearance could be confirmed. These observations suggest a better dispersion of the fluorinated COF with the Matrimid matrix, which agrees with the gas separation performance.

In addition, SEM images were taken of the top surface of the MMMs (Figure S26). As expected, more particles are visible on the surface at higher loadings, so that complete sedimentation of the COF particles can be excluded. The distribution of the filler HHU-COF-2 in the MMMs was investigated by fluorine element mapping using SEM-EDX (Figure S27). Due to the inaccuracy of EDX measurements for elements lighter than fluorine, these results should be considered critically. However, it can be seen that the COF particles are present throughout the whole membrane cross-sections.

The mechanical stability of the MMMs was investigated by determining the tensile strength. The maximum applied force until fracture of the respective MMM is summarized in Table S7. As expected, the pure Matrimid membrane exhibits the highest tensile strength of 91 MPa. For the MMMs with 8 and 16 wt% filler, values between 74 and 79 MPa were obtained. High loading of 24 wt% COF resulted in a reduction in the mechanical stability to below 60 MPa.

### 3.3. Gas Permeability and Selectivity

**Figure 8:**
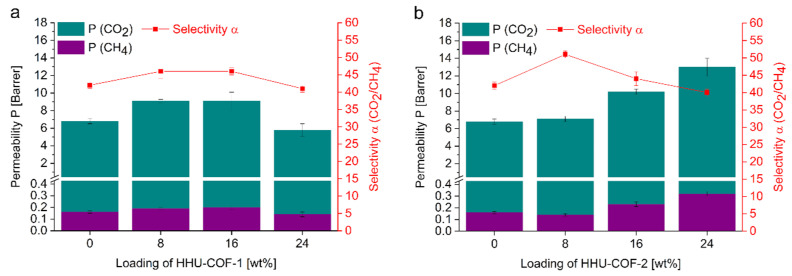
CO_2_ and CH_4_ permeabilities (*P*) and CO_2_/CH_4_ selectivity(α) for (**a**) HHU-COF-1/Matrimid and (**b**) HHU-COF-2/Matrimid MMMs.

The membrane performance of the two systems, HHU-COF-1/Matrimid and HHU-COF-2/Matrimid (Table 2; Figure 8), was investigated with respect to CO_2_/CH_4_ separation. The pure membranes and the membranes with 8, 16, and 24 wt% filler content were prepared in duplicate. Final permeability *P* was calculated with the thickness of the membranes given in Table S6. The CO_2_ permeability of the 8 and 16 wt% HHU-COF-1/Matrimid MMMs increased to 9.1 Barrer compared to the neat polymer membrane with 6.8 Barrer. This was accompanied by a moderate CO_2_/CH_4_ selectivity increase from 42 to 46. However, this increase from 6.8 to 9.1 Barrer is not very significant when compared with other porous organic framework MMMs (vide infra). With an increase in the filler content to 24 wt%, the permeability and selectivity decreased again to the approximate value of the neat Matrimid membrane, in line with the sedimentation noted from SEM. The HHU-COF-2-based MMMs showed a continuous enhancement in CO_2_ permeability upon increasing the filler content from 8 to 24 wt%. The CO_2_/CH_4_ selectivity was raised from 42 for neat Matrimid to 51 for the 8 wt% MMM. For the 16 and 24 wt% MMMs, the selectivity was back at the level of the pure polymer. The lower membrane efficiency of the HHU-COF-1-based membranes in comparison to HHU-COF-2 as filler material could be related to the blocking of the pores by (inter)penetrating polymer chains as well as the aggregation of the filler particles. A lower filler content of HHU-COF-1 seems to be recommended to achieve the optimum membrane performance for this system. For HHU-COF-2 as a dispersed phase, the known trade-off relationship between (high/low) permeability and (low/high) selectivity is seen as in the Robeson plots [43,44]. It was possible to consistently increase permeability with increasing filler content, which at the same time led to a decrease in selectivity. Overall, the fluorinated material showed better compatibility with the Matrimid matrix and led to a better separation performance for the gas pair CO_2_/CH_4_.

For a better understanding and classification of the permeation results, the fractional free volume (FFV) was calculated, and a comparison with permeability models was drawn. For the density (1.20 g/cm^3^) [45] and the FFV (0.167) [46] of Matrimid, the values reported in the literature were applied. The FFV of HHU-COF-1 and HHU-COF-2 was obtained by multiplying pore volume and density (from Equation (5)) and was calculated to be 0.558 and 0.560, respectively. The FFV of a MMM is the sum of the FFV of the polymer multiplied by the volume fraction of the continuous phase $\phi_{c}$ and the FFV of the filler material multiplied by the volume fraction of the dispersed phase $\phi_{d}$, as given in Equation (8):

(8) $$FFV=FFV_{polymer}*\phi_{c}+FFV_{filler}*\phi_{d}$$

**Figure 9:**
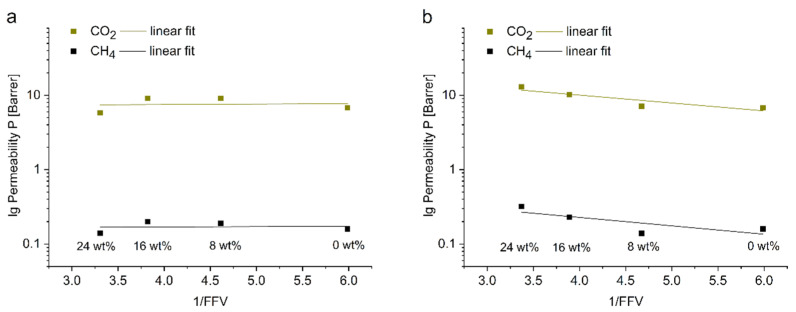
Lg permeability versus 1/FFV for 0 to 24 wt% (**a**) HHU-COF-1 and (**b**) HHU-COF-2 as dispersed phases in Matrimid MMMs.

The FFV does not contain the interface (void) volume. The logarithm of the CO_2_ and CH_4_ permeability versus 1/FFV for 0 to 24 wt% HHU-COF-1 and HHU-COF-2 as dispersed phases in Matrimid MMMs is plotted in Figure 9. Relating the free volume to the CO_2_ and CH_4_ permeability, HHU-COF-1 shows only a small increase up to a content of 16 wt%. For HHU-COF-1/Matrimid, the permeability of the 24 wt% MMM corresponds to the neat polymer (within experimental error) (Table 2). In turn, the actually available FFV of the 24 wt% MMM would only be that of the neat polymer. Thus, the theoretically calculated free volume from the filler is not available for permeation due to its aggregation and blocking or filling of the filler pores by polymer chains, as becomes clear when the FFV is put in relation to the permeability. For HHU-COF-2, lg*P* increases as 1/FFV decreases (i.e., when the amount of filler is increased) as expected. This is relatively more pronounced at high filler contents since the FFV of the filler material (0.560) is higher than that of the polymer (0.167).

To better assess the filler volume-dependent CO_2_ permeability increase, a comparison with the Maxwell model was carried out [47]. At first, it should be noted that this model describes an ideal particle distribution of the filler in the polymer. It also assumes spherical particles with a volume fraction of the dispersed filler phase $\phi_{d}$ up to 0.2. The simplified Maxwell model for the case that the permeability of the dispersed phase, *P_d,_* is much higher than the permeability of the pure continuous (polymer) phase, *P_c,_* is shown in Equation (9), where *P_eff_* stands for the permeability of the MMMs:

(9) $$\frac{P_{eff}}{P_{c}}=\frac{1+2\phi_{d}}{1-\phi_{d}}$$

**Figure 10:**
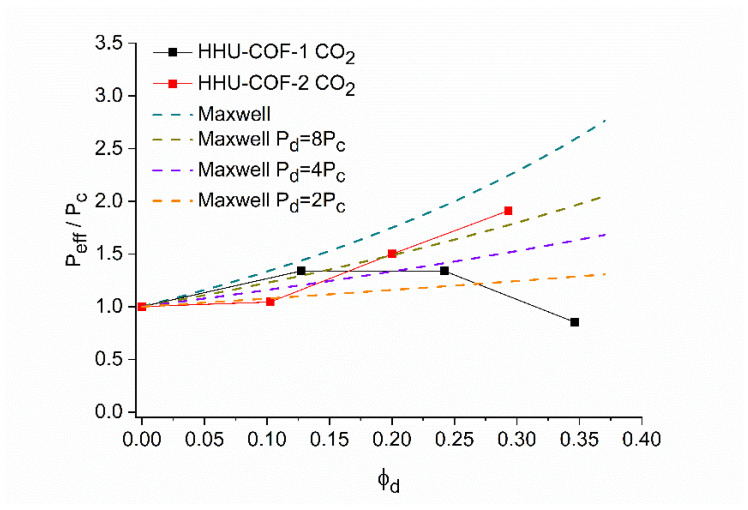
*P_eff_* /*P_c_* of the gas CO_2_ versus $\phi_{d}$ for 0 to 24 wt% HHU-COF-1 and HHU-COF-2 as dispersed phases in Matrimid matrices.

In addition, three further modified models were used, which assume *P_d_* = 8*P_c_*, *P_d_* = 4*P_c,_* and *P_d_* = 2*P_c._* Models that are applicable to higher $\phi_{d}$ [48,49] also show much higher values for *P_eff_* /*P_c_* as a function of $\phi_{d}$ and are not applicable for a comparison in this case. Figure 10 plots the ratio *P_eff_* /*P_c_* of the gas CO_2_ versus $\phi_{d}$ for the filler materials HHU-COF-1 and HHU-COF-2 in the polymer Matrimid in comparison to the Maxwell model and the modified models. The higher volume fractions of HHU-COF-1 over HHU-COF-2 are due to the lower density of the HHU-COF-1 for equivalent mass percentages. For the HHU-COF-1 MMMs, a low filler efficiency is confirmed with respect to CO_2_ permeability. Only the 8 wt% MMM shows an increase in permeability, which agrees with the Maxwell model for *P_d_* = 8*P_c_*. The 16 wt% HHU-COF-1 MMM already exceeds the filler volume fraction of 0.2, for which the Maxwell model is applicable. As the filler content of HHU-COF-2 increases, the *P_eff_* /*P_c_* ratio increases significantly higher. The 8 wt% MMM agrees with the model prediction for *P_d_* = 2*P_c_* and the 16 wt% MMM with *P_d_* = 8*P_c_*. The 24 wt% MMM represents the most efficient membrane in terms of CO_2_ permeability. With a *P_eff_* /*P_c_* ratio of 1.9, it exceeds the Maxwell model for *P_d_* = 8*P_c_*.

A comparison with literature values of Matrimid-based MMMs for CO_2_/CH_4_ separation shows that the performance of the 8 wt% HHU-COF-1 MMM with a CO_2_ permeability of 9.1 Barrer agrees with comparable systems, such as ACOF-1 [31] or CTF-fluorene [50] in Matrimid. The system with 8 wt% of the azine-linked structure ACOF-1 in Matrimid achieved a CO_2_ permeability value of 9.6 Barrer, and the corresponding weight percentage of CTF-fluorene in Matrimid increased the CO_2_ permeability to 9.2 Barrer. The application of 8 wt% of a biphenyl-based CTF with a larger pore volume resulted in a value of 12.0 Barrer [36]. At this point, it is once again confirmed that increasing the pore size is only beneficial up to a certain level. A comparison with different filler materials in matrices such as 6FDA-DAM [51], Pebax [52], and PIM-1 [32,53] can be found in Table S9. In contrast, the higher filler amounts of HHU-COF-2 showed a much larger increase in CO_2_ permeability. Compared to the pure Matrimid membrane, the CO_2_ permeability for the 24 wt% MMM was increased by 91% to 13.0 Barrer.

To test the long-term stability of the MMMs, we performed permeability measurements again after one year (Table S8). All 12 MMMs were covered but stored without further protection at an average temperature of 20 °C in ambient air. The 8 and 16 wt% HHU-COF-1/MMMs showed reduced CO_2_ permeability with values of 8.6 and 8.0 Barrer, respectively. The 24 wt% MMM proved to be stable in terms of CO_2_ permeability, but the CO_2_/CH_4_ selectivity was reduced to 28, so the MMM can be considered defective. The HHU-COF-2 MMMs not only showed the best results in terms of initial membrane performance but also exhibited consistent CO_2_ permeability after one year of storage. CO_2_ permeability of 7.1 (8 wt%), 10.5 (16 wt%), and 12.9 Barrer (24 wt%) was measured with CO_2_/CH_4_ selectivity between 44 and 45 after this one year.

In summary, from the permeability and FFV values, it is evident that the high theoretical filler FFV does not contribute to a significant increase in permeability. Evidently, the pore openings in HHU-COF-1 and HHU-COF-2 are too large (cf. Scheme 1) so that they allow for (inter)penetration of polymer chains that then drastically decrease this fractional free volume contribution from the filler. The higher increase in permeability for HHU-COF-2 over HHU-COF-1 for the 16 and 24 wt% MMMs indicates that HHU-COF-2 with the fluorinated linker has a higher filler contribution. This is explained by a hindrance of the (inter)penetration of the non-fluorinated polymer chains into the fluorous HHU-COF-2 inner pores, akin to fluorous chemistry with the lower affinity or even separation of perfluorinated compounds from non-fluorinated phases.

## 4. Conclusions

Two imine-linked COFs were synthesized via a Schiff base reaction applying 1,3,5-tris-(4-aminophenyl)triazine as an amine compound. 4,4′-biphenyldicarboxaldehyde and the corresponding octafluoro derivative were chosen as linkers to form HHU-COF-1 and HHU-COF-2. The successful formation of the materials was confirmed by ^13^C and ^19^F CP MAS NMR, IR, XPS, and elemental analysis. Both materials are crystalline, and the synthesis with the biphenyl-based linkers led to high BET surface areas of 2352 m^2^/g for HHU-COF-1 and 1356 m^2^/g for HHU-COF-2. Moreover, both syntheses were successfully carried out on a larger scale in order to apply the products as filler materials in Matrimid-based MMMs for CO_2_/CH_4_ separation, where the fluorinated COF led to the better membrane performance with a CO_2_ permeability increase from 6.8 to 13.0 Barrer for the 24 wt% MMM.

## Figures and Tables

**Table 1:** Elemental analysis of HHU-COF-1 and HHU-COF-2.

COF	C (wt%)	H (wt%)	N (wt%)
HHU-COF-1 Calculated	81.93	4.42	13.65
HHU-COF-1	81.09	4.27	13.17
HHU-COF-2 Calculated ^a^	60.66	1.82	10.11
HHU-COF-2 ^b^	60.94	1.93	10.35

^a^ This leaves calculated 27.41 wt% for fluorine. ^b^ 26.78 wt% difference to 100 wt%.

**Table 2:** Gas permeabilities (P) and mixed-gas selectivity factors (α) of the pristine Matrimid membrane and COF/Matrimid MMMs ^1^.

Filler Material	Filler Content (wt%)	P CO_2_ (Barrer)	P CH_4_ (Barrer)	α CO_2_/CH_4_
-	0	6.8 ± 0.3	0.16 ± 0.01	42 ± 1
HHU-COF-1	8	9.1 ± 0.2	0.19 ± 0.01	46 ± 2
	16	9.1 ± 1.0	0.20 ± 0.02	46 ± 1
	24	5.8 ± 0.7	0.14 ± 0.02	41 ± 1
HHU-COF-2	8	7.1 ± 0.3	0.14 ± 0.01	51 ± 1
	16	10.2 ± 0.3	0.23 ± 0.02	44 ± 2
	24	13.0 ± 1.0	0.32 ± 0.02	40 ± 1

^1^ The errors for the permeability (P) and for the selectivity (α) were taken from the range of two to three measurements and include the upper and lower value.

## Data Availability

The data presented in this study are available on request from the corresponding author.

## Supplementary Materials

The following supporting information can be downloaded at: https://www.mdpi.com/article/10.3390/ma15082807/s1, Scheme S1. Schematic formation of TRITER-1 (=SCF-HCOF-1) and SCF-FCOF-1 from TAPT and TA or TFTA, respectively. (TAPB = 1,3,5-tris(4-aminophenyl) benzene, TAPT = 2,4,6-tris(4-aminophenyl)-1,3,5-triazine, TA = terephthalaldehyde, TFTA = 2,3,5,6-tetrafluoroterephthaldehyde). The edge-edge distance was taken from the literature of SCF-FCOF-1 and -2; Figure S1. ^19^F NMR spectrum of perfluorinated nitrile 1; Figure S2. ^1^H NMR spectrum of perfluorinated aldehyde 2; Figure S3. ^19^F NMR spectrum of perfluorinated aldehyde 2; Figure S4. ^13^C{^1^H} NMR spectrum of perfluorinated aldehyde 2; Figure S5. EI-MS (at 80 °C) spectrum of perfluorinated aldehyde 2; Figure S6. AT-IR spectrum of perfluorinated aldehyde 2; Figure S7. XPS survey spectra of HHU-COF-1 and HHU-COF-2; Figure S8. High-resolution XPS spectra of the C1s region and N1s region of HHU-COF-1; Figure S9. High-resolution XPS spectra of the C1s region and N1s region of HHU-COF-2; Figure S10. High-resolution XPS spectra of the F1s region of HHU-COF-2; Figure S11. SEM images of HHU-COF-1; Figure S12. SEM image of HHU-COF-2; Figure S13. CO_2_ and CH_4_ sorption isotherms at 273 K of HHU-COF-1 and HHU-COF-2, respectively; Figure S14. IAST selectivities of HHU-COF-1 and HHU-COF-2 for a binary (50:50; *v*:*v*) mixture of the gases CO_2_/CH_4_ at 273 K; Figure S15. First derivative of TGA curves of HHU-COF-1 and HHU-COF-2. Measurement under nitrogen atmosphere with a heating rate of 5 K/min; Figure S16. Correlation of the 2theta (2θ) values from the powder X-ray diffractograms in Figure 5 in the main text with the reflection planes and the d spacing according to the Bragg equation n λ = 2d sinθ or d = nλsinθ with λ = 1.5406 Å and n = 1. Note that the edge-edge distances a and the triazine-centroid triazine-centroid (tz-tz) distances along the edge derived therefrom as (a/2)/cos30° were determined from the most intense and, thus, most accurately measurable (100) reflexes in the powder-X-ray diffractograms of HHU-COF-1 and -2; Figure S17. Images of HHU-COF-1 and HHU-COF-2; Figure S18. IR spectra of HHU-COF-1 (larger scale) and HHU-COF-2 (larger scale); Figure S19. Nitrogen sorption isotherm and pore size distribution calculated with slit pore, NLDFT equilibrium model of HHU-COF-1 (larger scale); Figure S20. Nitrogen sorption isotherm and pore size distribution calculated with slit pore, NLDFT equilibrium model of HHU-COF-2 (larger scale); Figure S21. TGA curves of HHU-COF-1 (larger scale) and HHU-COF-2 (larger scale). Acquired under nitrogen atmosphere with a heating rate of 5 K/min; Figure S22. PXRD pattern of HHU-COF-1 (larger scale) and HHU-COF-2 (larger scale); Figure S23. Schematic preparation of the pure Matrimid membrane and MMMs, using the 16 wt% HHU-COF-2 MMM as an example; Figure S24. Preparation of membranes by solution casting: casting the solution, drying, cutting with a scalpel, and removing the membrane; Figure S25. Setup for CO_2_/CH_4_ mixed-gas separation measurements; Figure S26. Top-surface SEM images of HHU-COF-1/Matrimid with 8, 16, and 24 wt% filler and HHU-COF-2/Matrimid MMMs with 8, 16, and 24 wt% filler; Figure S27. SEM images of HHU-COF-2/Matrimid MMMs with 8, 16, and 24 wt% filler and associated fluorine elemental mapping; Figure S28. IR spectra of TRITER-1 and SCF-FCOF-1; Figure S29. SEM image of SCF-FCOF-1 and associated fluorine elemental mapping; Figure S30. Nitrogen sorption isotherm and pore size distribution calculated with slit pore, NLDFT equilibrium model of TRITER-1; Figure S31. Nitrogen sorption isotherm and pore size distribution calculated with slit pore, NLDFT equilibrium model of SCF-FCOF-1; Figure S32. TGA curves of TRITER-1 and SCF-FCOF-1. Acquired under nitrogen atmosphere with a heating rate of 5 K/min; Figure S33. PXRD pattern of TRITER-1 and SCF-FCOF-1; Figure S34. Images of TRITER-1 and SCF-FCOF-1; Table S1. at% and wt% of the elements in HHU-COF-1 and HHU-COF-2 obtained from XPS survey spectra; Table S2. EDX analysis of HHU-COF-2; Table S3. Comparison of CO_2_ uptake and BET surface area of imine-linked/azine COFs; Table S4. Parameters for LAI and Toth fitting; Table S5. Elemental analysis of HHU-COF-1 (larger scale) and HHU-COF-2 (larger scale); Table S6. Average thickness of MMMs; Table S7. Tensile strength of pure Matrimid and MMMs. Table S8. Gas permeabilities (P) and mixed-gas selectivity factors (α) for COF/Matrimid MMMs when stored for one year under ambient conditions; Table S9. Comparison of CO_2_ permeability and CO_2_/CH_4_ selectivity for COFs/CTFs as porous filler materials in different polymer MMMs; Table S10. Elemental analysis of TRITER-1 and SCF-FCOF-1; Table S11. EDX analysis of SCF-FCOF-1. References [54,55,56,57,58,59] cited in Supplementary Materials.
