# Supplementary material for: Synthesis and Characterization of a Crystalline Imine-Based Covalent Organic Framework with Triazine Node and Biphenyl Linker and Its Fluorinated Derivate for CO2/CH4 Separation
Source: Materials (Basel). 2022 Apr 11;15(8):2807. doi: 10.3390/ma15082807 (PMC9031922; doi:10.3390/ma15082807)
Supplement: Supplementary file 1 [file materials-15-02807-s001.zip › materials-1638974-supplementary.pdf]

Supplementary Materials

Synthesis and Characterization of a Crystalline Imine-Based Covalent Organic Framework with Triazine Node and Biphenyl Linker and Its Fluorinated Derivate for CO_2/CH_4 Separation

Stefanie Bügel [1], Malte Hähnel [1], Tom Kunde [2], Nader de Sousa Amadeu [3], Yangyang Sun [1], Alex Spieß [1], Thi Hai Yen Beglau [1], Bernd M. Schmidt [2,*] and Christoph Janiak [1,*]

1 Institut für Anorganische Chemie und Strukturchemie, Heinrich-Heine-Universität Düsseldorf, 40204 Düsseldorf, Germany; stefanie.buegel@hhu.de (S.B.); mahae107@hhu.de (M.H.); yasun100@hhu.de (Y.S.); alex.spiess@hhu.de (A.S.); beglau@hhu.de (T.H.Y.B.)

2 Institut für Organische Chemie und Makromolekulare Chemie, Heinrich-Heine-Universität Düsseldorf, 40204 Düsseldorf, Germany; kunde@hhu.de

3 Bundesanstalt für Materialforschung und -Prüfung, Fachbereich 6.3 (Strukturanalytik), 12489 Berlin, Germany; nader.amadeu@bam.de

* Correspondence: bernd.schmidt@hhu.de (B.M.S.); janiak@hhu.de (C.J.)

Table of Contents

1. Synthetic procedure for 2,2',3,3',5,5',6,6'-octafluoro-4,4'-biphenyldicarboxaldehyde 3
    1.1. Synthesis of 1,1’-(dicyano)-2,2’,3,3’,5,5’,6,6’-octafluoro-4,4’-biphenyl (1) 3
    1.2. Synthesis of 2,2',3,3',5,5',6,6'-octafluoro-4,4'-biphenyldicarboxaldehyde (2) 3
2. Characterization of HHU-COF-1 and HHU-COF-2 7
    2.1. X-ray photoelectron spectroscopy (XPS) 7
    2.2. Scanning electron microscopy (SEM) 9
    2.3. Energy dispersive X-ray spectroscopy (SEM-EDX) 9
    2.4. CO_2- and CH_4-sorption 10
    2.5. Ideal adsorbed solution theory (IAST) selectivities 12
    2.6. Thermogravimetric analysis (TGA) 13
    2.7. Correlation of 2theta values 13
    2.8. Images of HHU-COF-1 and HHU-COF-2 14
3. Characterization of HHU-COF-1 (larger scale) and HHU-COF-2 (larger scale) 14
    3.1. Infrared (IR) spectroscopy 14
    3.2. Elemental analysis 14
    3.3. N_2-sorption 15
    3.4. TGA 16
    3.5. Powder X-Ray diffraction (PXRD) 16
4. Preparation and characterization of MMMs 17
    4.1. Schematic preparation of the pure polymer membrane and MMMs 17
    4.2. Casting procedure 17
    4.3. Set-up for CO_2/CH_4 mixed gas separation measurements 18
    4.4. Membrane thickness 18
    4.5. SEM images of membrane surfaces 19
    4.6. SEM-EDX of HHU-COF-2/Matrimid MMMs 19
    4.7. Tensile strength 20
    4.8. Long-term stability of MMMs 20
    4.9. Comparison of membrane performance 21
5. Synthesis and characterization of TRITER-1 (= SCF-HCOF-1) and SCF-FCOF-1 22
    5.1. Materials and Synthesis 22
    5.2. IR spectroscopy 24
    5.3. Elemental analysis 24
    5.4. SEM-EDX 24
    5.5. N_2-sorption 25

5.6. TGA 26
5.7. PXRD 26
5.8. Images of TRITER-1 and SCF-FCOF-1 27
6. References 28

1. Synthetic procedure for 2,2',3,3',5,5',6,6'-octafluoro-4,4'-biphenyldicarboxaldehyde

1.1. Synthesis of 1,1'-(dicyano)-2,2',3,3',5,5',6,6'-octafluoro-4,4'-biphenyl (1)

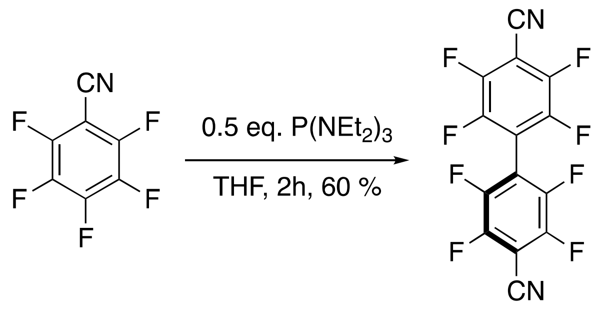

Pentafluorobenzonitrile (8.10 mL, 12.30 g, 64 mmol) was dissolved in 200 mL of dry THF and Tris(diethyl-amino)phosphine (8.16 g, 0.52 eq.) were added dropwise under nitrogen and stirring. The mixture was allowed to stir for 2 hours at room temperature. After the TLC indicated the complete consumption of the starting material, the reaction was quenched *via* the addition of 40 mL 2N hydrochloric acid. The mixture was extracted with diethyl ether (3 x 50 mL) and was dried over anhydrous magnesium sulphate. After evaporation of the solvent the oily residue was left to crystallize to yield faint yellow crystalline blocks. yield: 6.68 g; 19 mmol; 60%

The analytical data was in accordance with the literature [1].

^{19}F NMR(282 MHz, $CDCl_3$): δ -129.65 (d, Ar-**F_A**), -133.47 (d, Ar-**F_B**)

1.2. Synthesis of 2,2',3,3',5,5',6,6'-octafluoro-4,4'-biphenyldicarboxaldehyde (2)

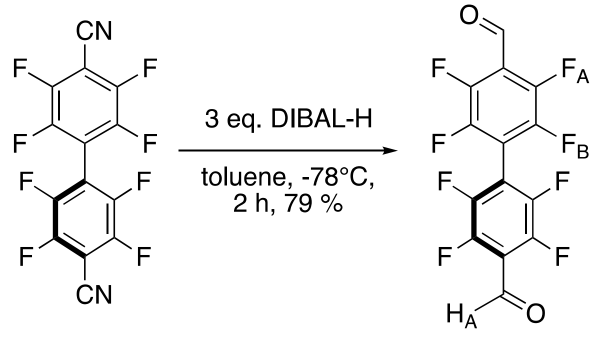

1,1'-(Dicyano)-2,2',3,3',5,5',6,6'-octafluoro-4,4'-biphenyl (2.00 g, 5.74 mmol) was dissolved in toluene (50 mL) and the resulting solution was thoroughly degassed *via* purging with argon for 15 minutes. A solution of diisobutylaluminium hydride (DIBAL-H) in toluene (1.5 M, 11.5 mL, 17.22 mmol) was added dropwise over a period of 30 minutes at -78 °C. After complete addition, the resulting mixture was stirred for additional 2 hours. The reaction was quenched by addition of 10 mL of ethyl acetate and 30 mL of 2N hydrochloric acid. The organic phase was separated and the aqueous phase was extracted with dichloromethane (2 x 100 mL).

The combined organic phases were dried over magnesium sulphate and the solvent was evaporated under reduced pressure to yield **2** as a colorless powder. yield: 1.6 g; 4.51 mmol; 79%

^{1}H NMR(300 MHz, $CDCl_3$): δ 10.39 (s, -C**H_A**O); **^{19}F NMR**(282 MHz, $CDCl_3$): δ -136.15 (m, Ar-**F_A**), -143.70 (m, Ar-**F_B**); **^{13}C{1H} NMR** (75 MHz, $CDCl_3$) δ 181.90 (s, Ar-**C**H_AO), 147.24 (d, *J* = 210.5 Hz, **C_{Ar}**-F_A), 143.76 (d, *J* = 207.0 Hz, **C_{Ar}**-F_B), 117.05 (t, *J* = 9.8 Hz, **C_{Ar}**-CH_AO), 111.89 (m, **C_4/$C_{4'}$**); **FT-IR (ATR):** $\tilde{\nu}$(cm^{-1}) = 2910.6 (w), 2358.9 (w), 2339.7 (w), 1712.8 (s), 1651.1 (m), 1575.8 (w), 1473.6 (s), 1408.0 (m), 1381.0 (m), 1357.9 (w), 1317.4 (w), 1298.1 (m), 1276.9 (s), 1114.9 (w), 1018.4 (s), 1003.0 (s), 989.5 (s), 956.7 (s), 914.3 (m), 800.5 (m), 721.4 (s); **EI-MS (80 °C):** calc. for $[C_{14}H_2F_8O_2–H]^+$ = 352.9843 m/z; found: 353.0 m/z (100%, $[M-H]^+$), 324.9 (27%, $[M-CHO]^+$), 297.0 m/z (26%, $[M-2(CHO)+H]^+$), 278.0 m/z (48%, $[M(297\ m/z)-F]^+$); **M_p:** 144.8-145.1 °C.

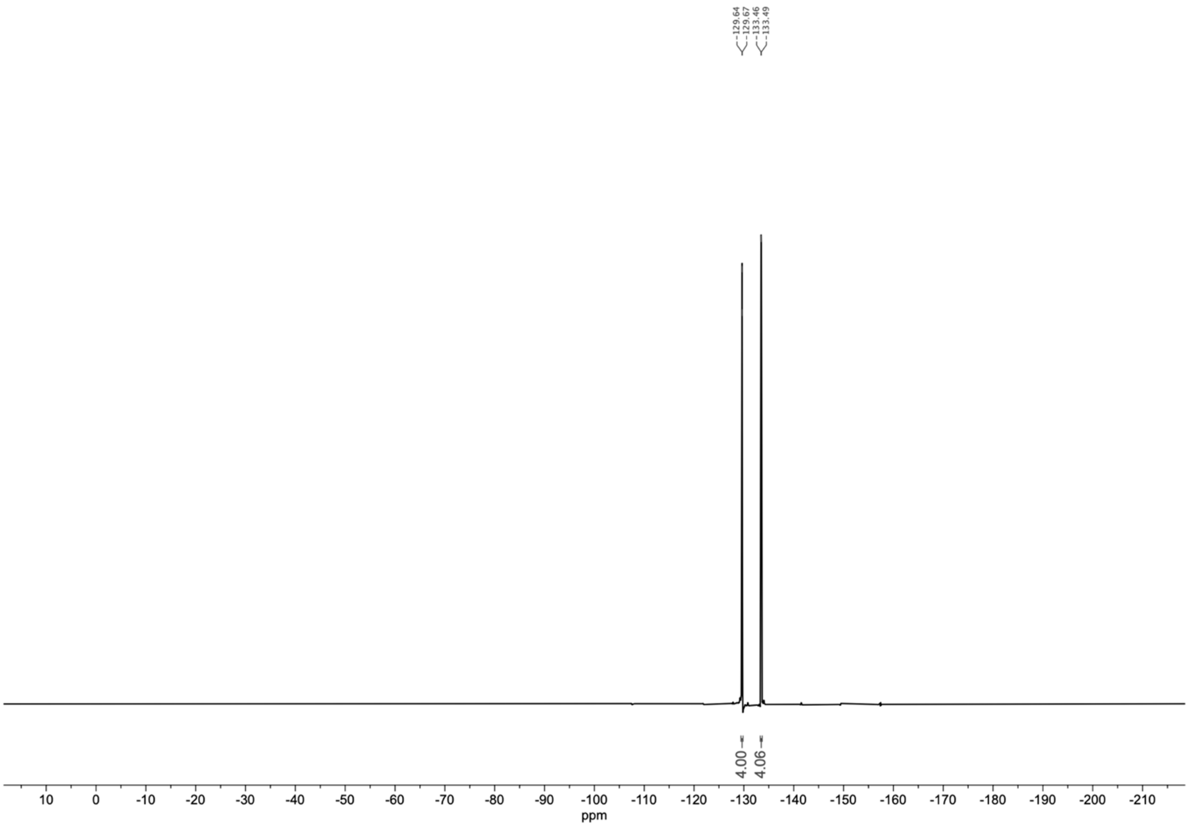

Figure S1. ^{19}F NMR spectrum of perfluorinated nitrile **1**.

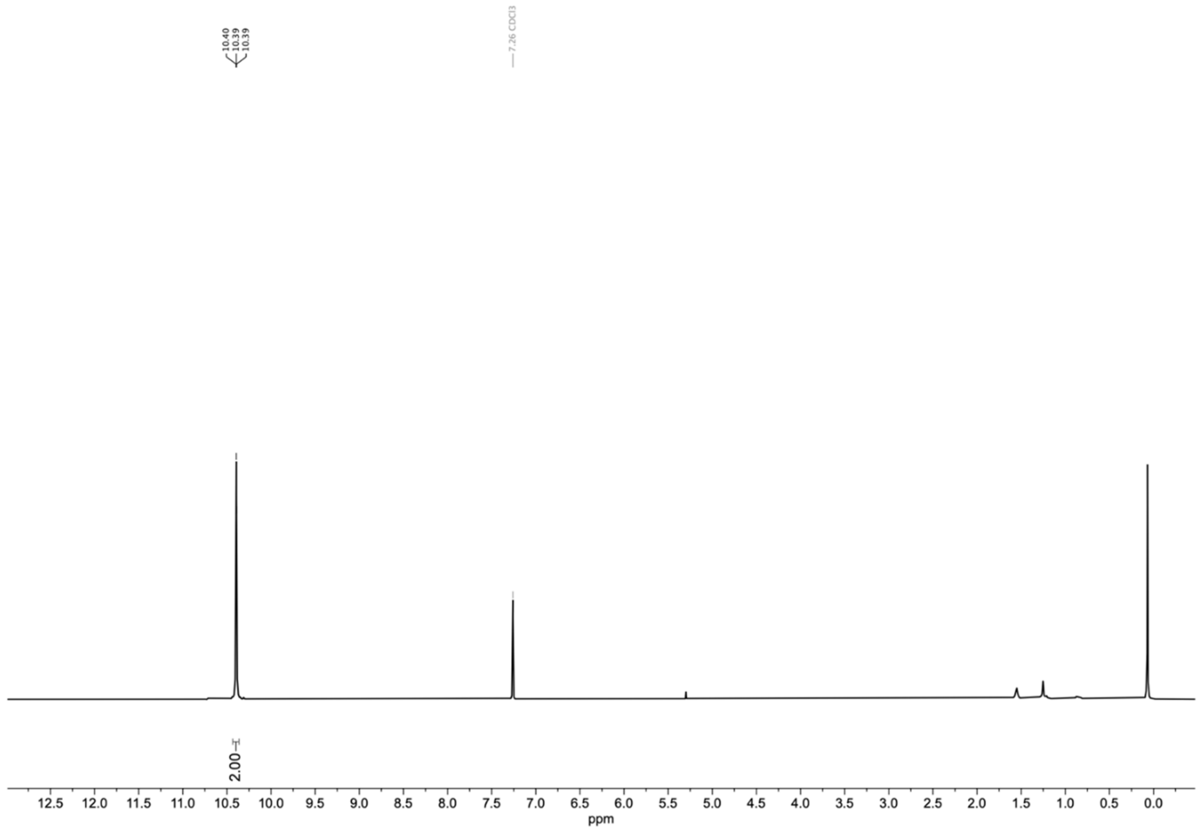

Figure S2. ^{1}H NMR spectrum of perfluorinated aldehyde **2**.

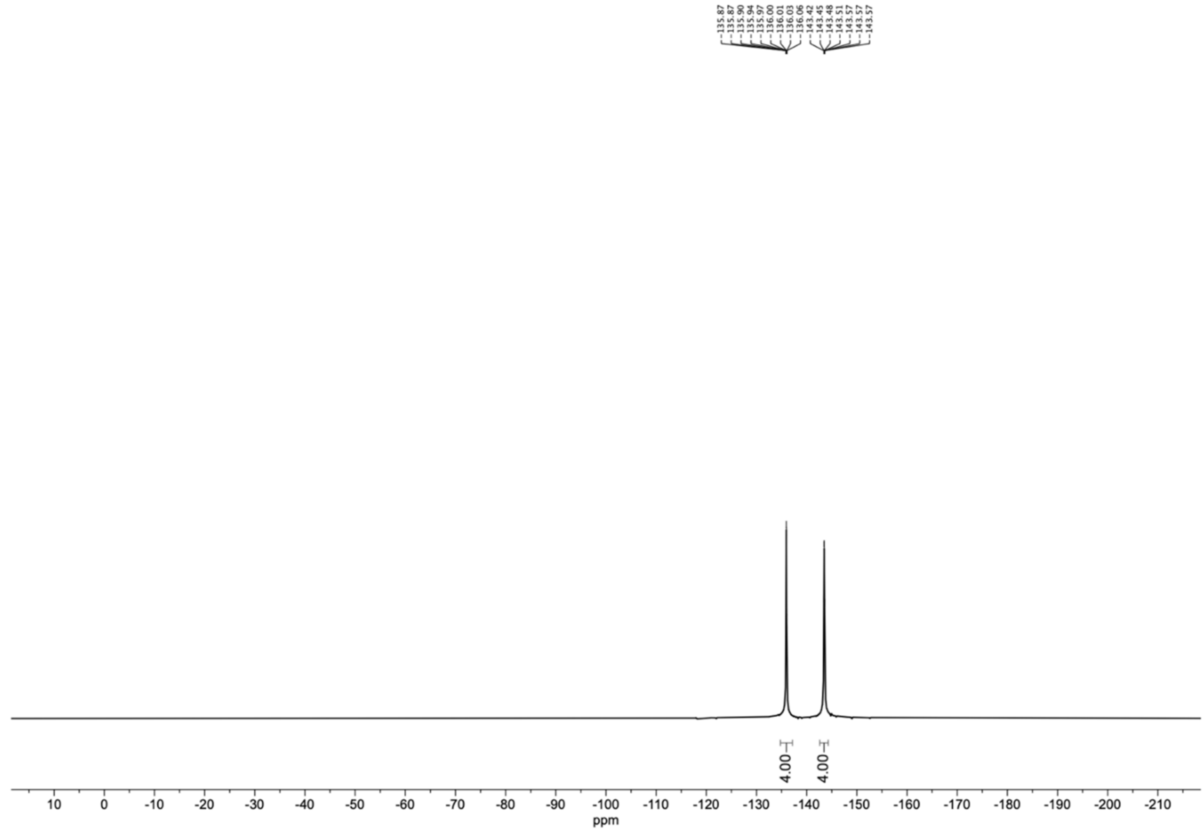

Figure S3. ^{19}F NMR spectrum of perfluorinated aldehyde **2**.

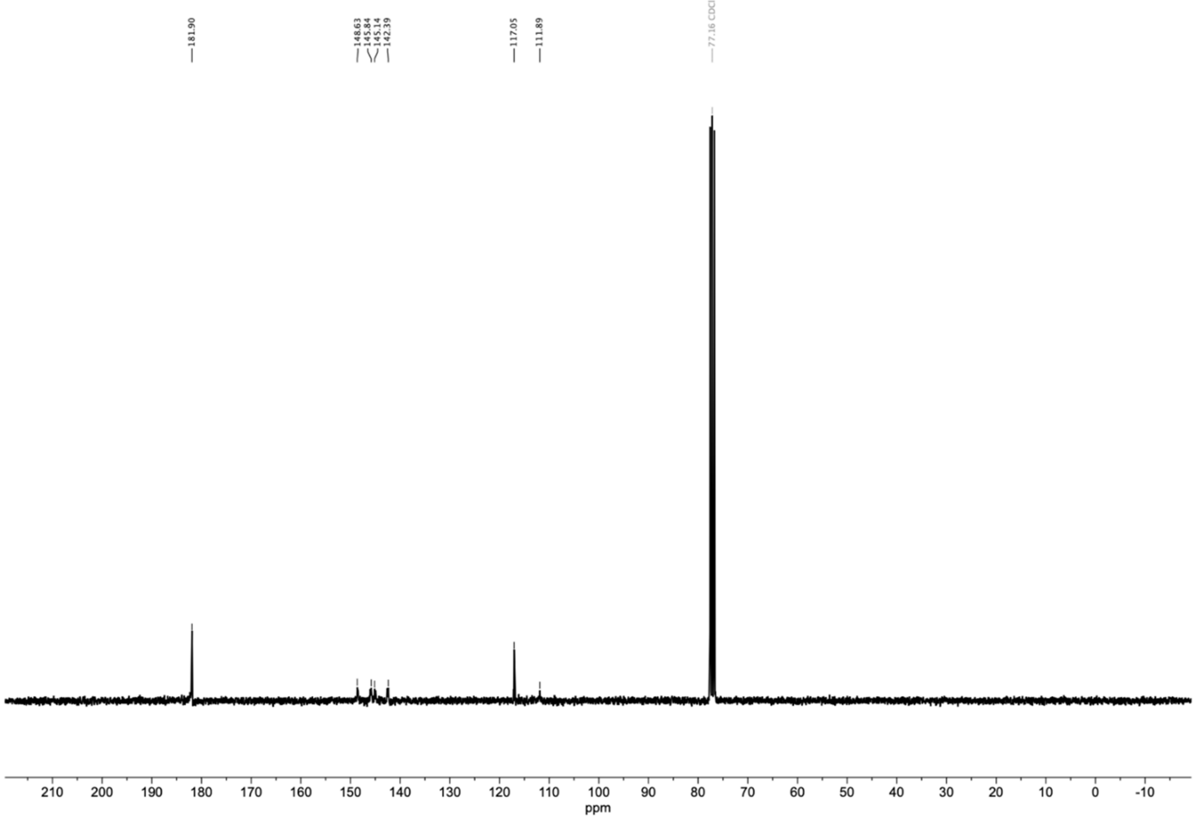

Figure S4. $^{13}C\{^{1}H\}$ NMR spectrum of perfluorinated aldehyde **2**.

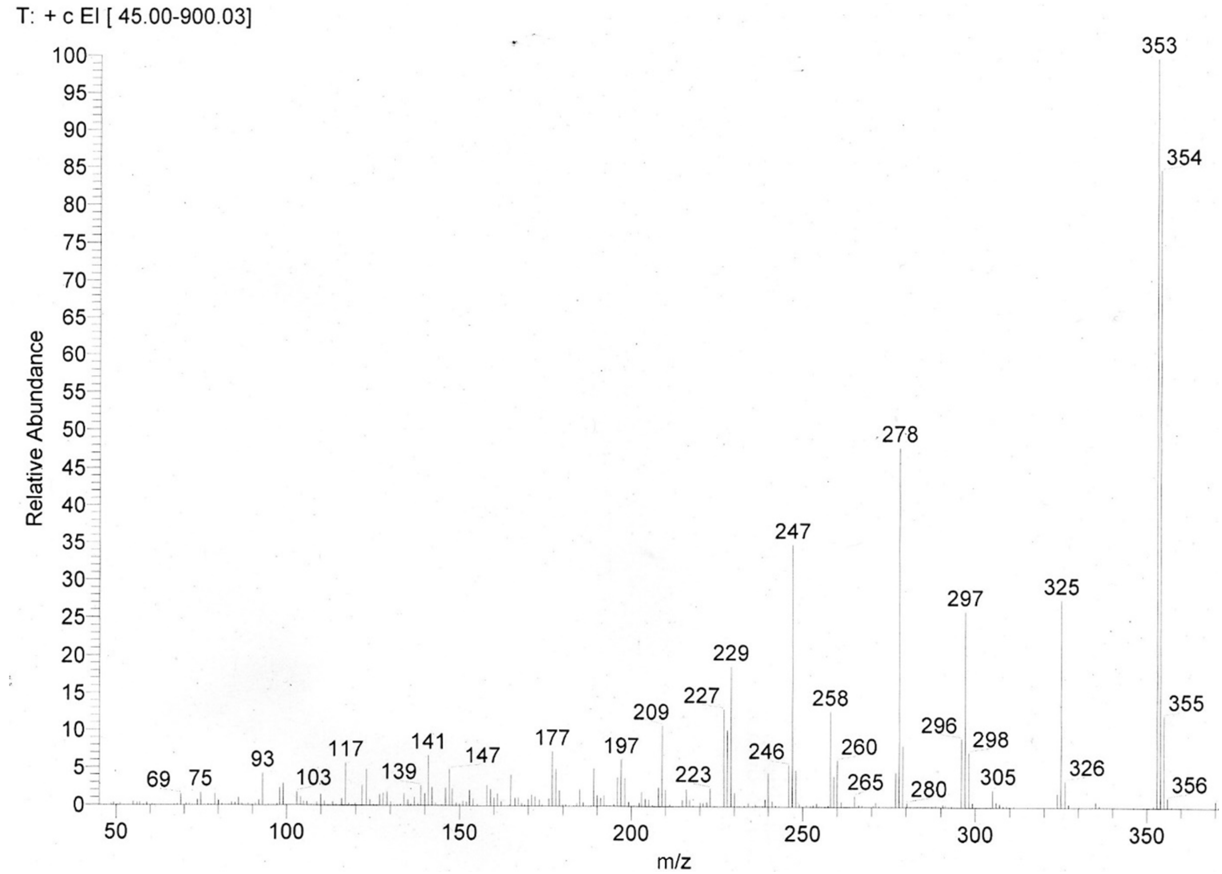

Figure S5. EI-MS (at 80 °C) spectrum of perfluorinated aldehyde **2**.

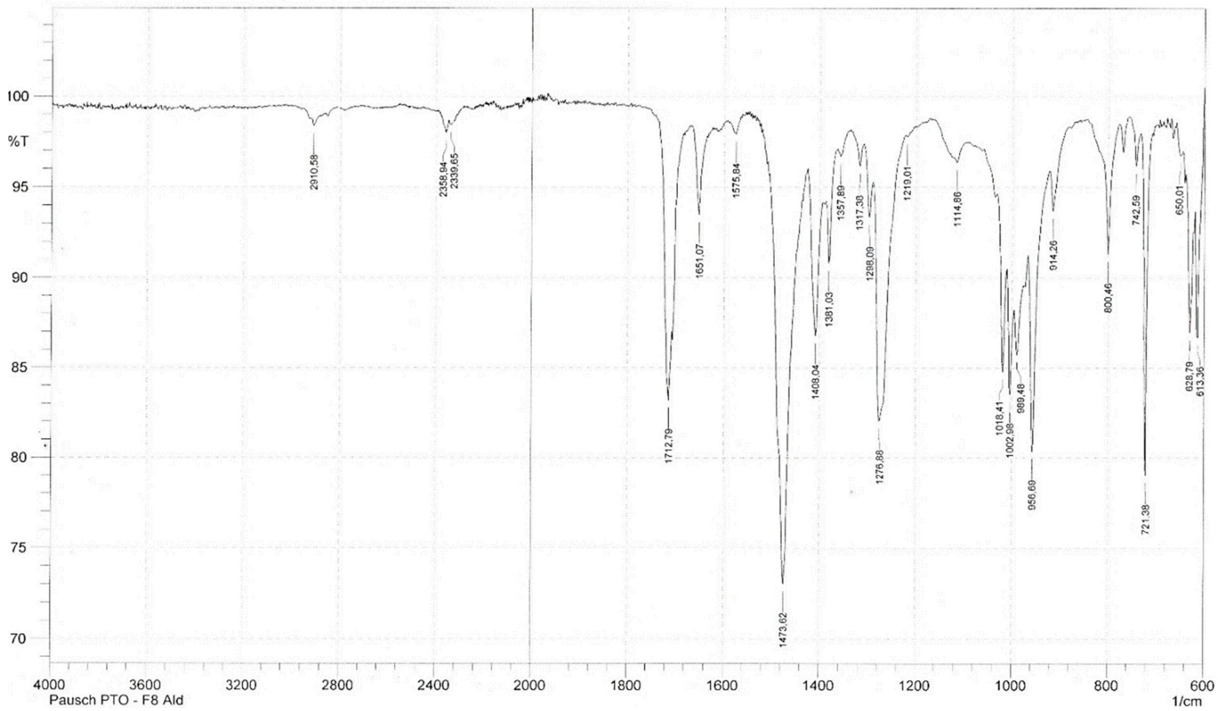

Figure S6. AT-IR spectrum of perfluorinated aldehyde **2**.

2. Characterization of HHU-COF-1 and HHU-COF-2

2.1. X-ray photoelectron spectroscopy (XPS)

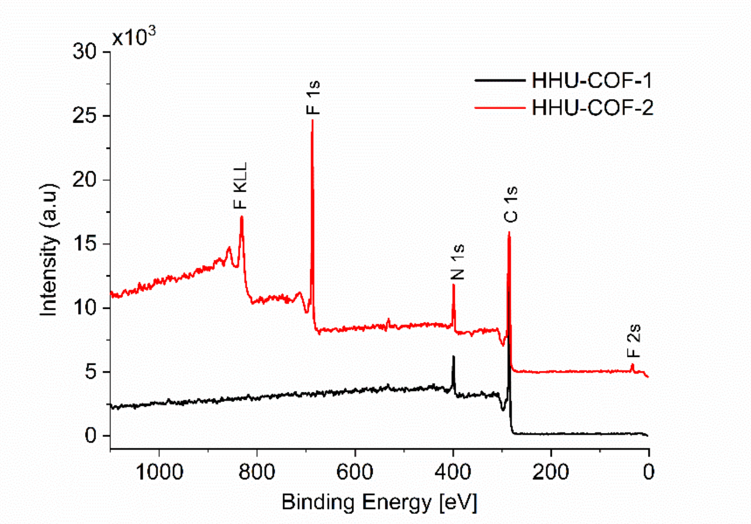

Figure S7. XPS survey spectra of HHU-COF-1 and HHU-COF-2.

Table S1. at% and wt% of the elements in HHU-COF-1 and HHU-COF-2 obtained from XPS survey spectra.

COF	C1s		N 1s		F1s	
	[at%]	[wt%]	[at%]	[wt%]	[at%]	[wt%]
HHU-COF-1	89.2	87.6	10.9	12.4	-	-
HHU-COF-2	70.8	62.7	9.8	10.1	19.5	27.3

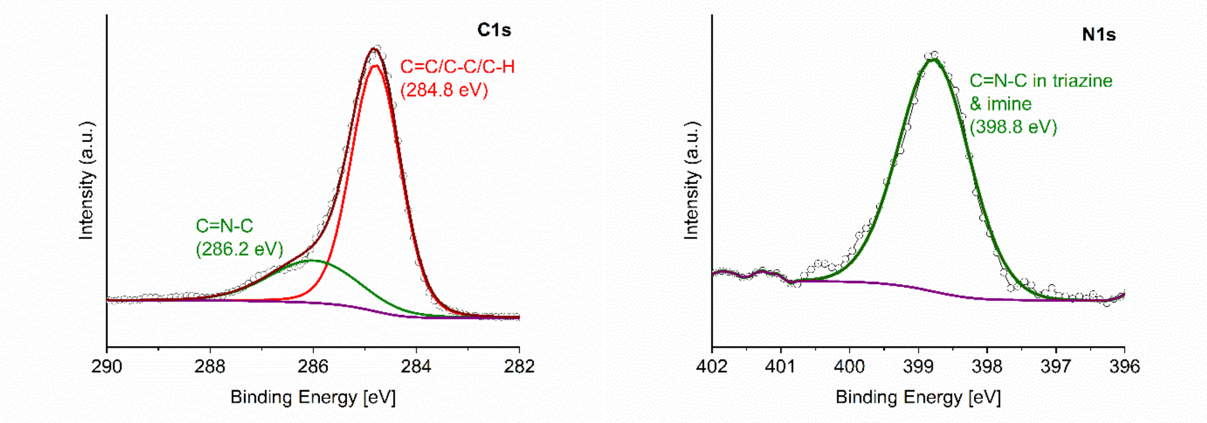

Figure S8. High-resolution XPS spectra of the C1s region (left) and N1s region (right) of HHU-COF-1.

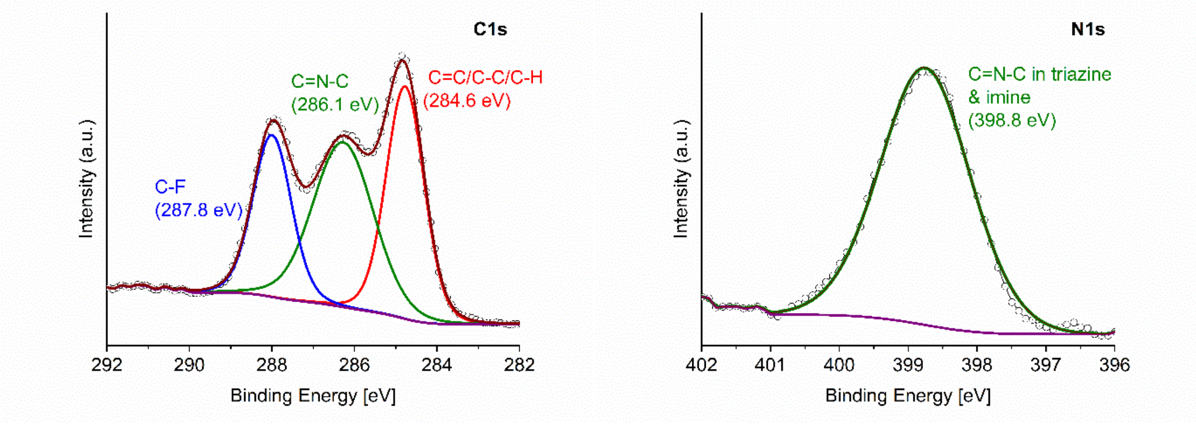

Figure S9. High-resolution XPS spectra of the C1s region (left) and N1s region (right) of HHU-COF-2.

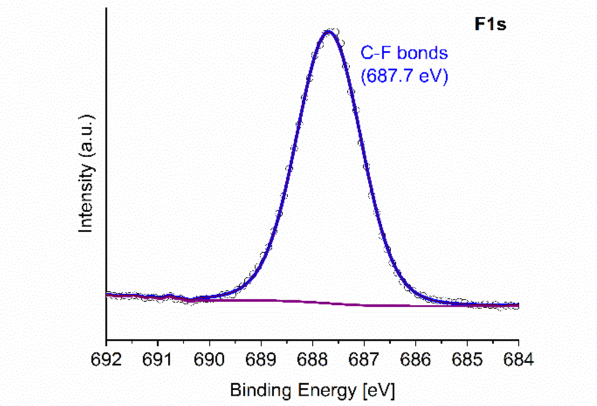

Figure S10. High-resolution XPS spectra of the F1s region of HHU-COF-2.

2.2. Scanning electron microscopy (SEM)

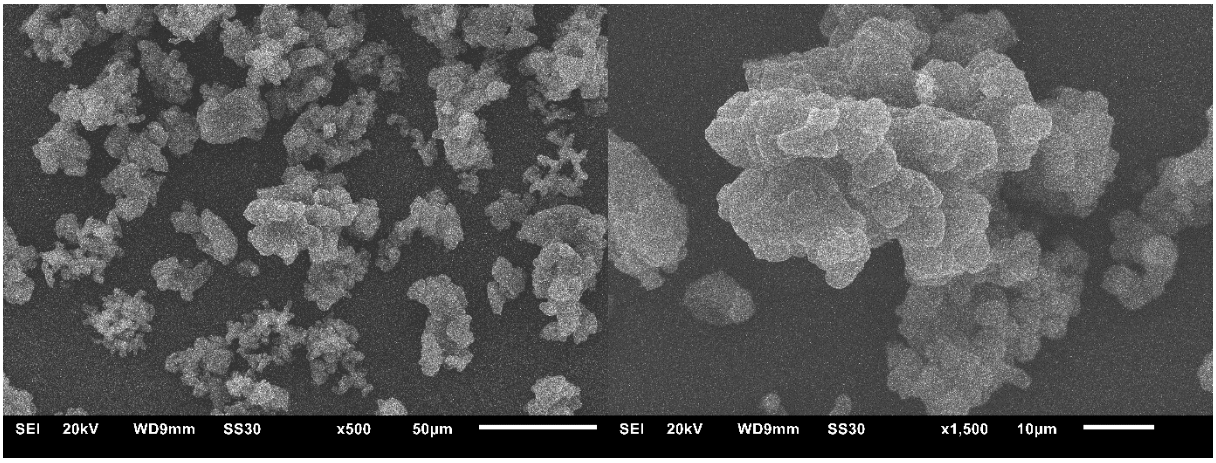

Figure S11. SEM images of HHU-COF-1.

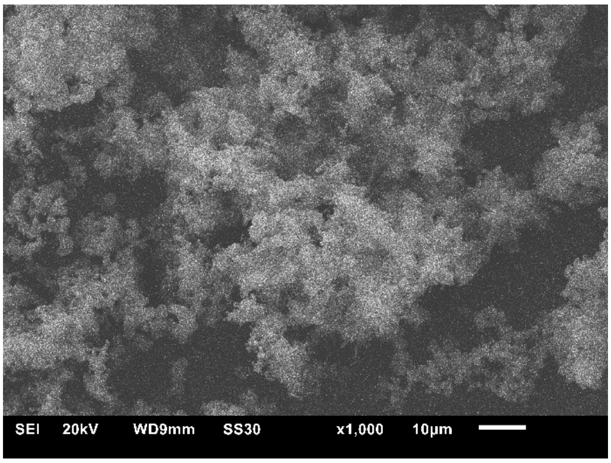

Figure S12. SEM image of HHU-COF-2.

2.3. Energy dispersive X-ray spectroscopy (SEM-EDX)

Table S2. EDX analysis of HHU-COF-2.

	C	H	F	N	O	Au	Cu	Zn	Sum
Ideal [wt%]	60.66	1.82	27.41	10.11	-	-	-	-	100.0
EDX [wt%][a]	63.1	-	16.3	7.3	3.5	7.7	1.2	0.9	100.0
EDX [wt%] with C,F,N	63.1	-	16.3	7.3	-	-	-	-	86.7
EDX [wt%][a] with C,F,N	72.8	-	18.8	8.4	-	-	-	-	100.0

[a] normalized

2.4. CO_2- and CH_4-sorption

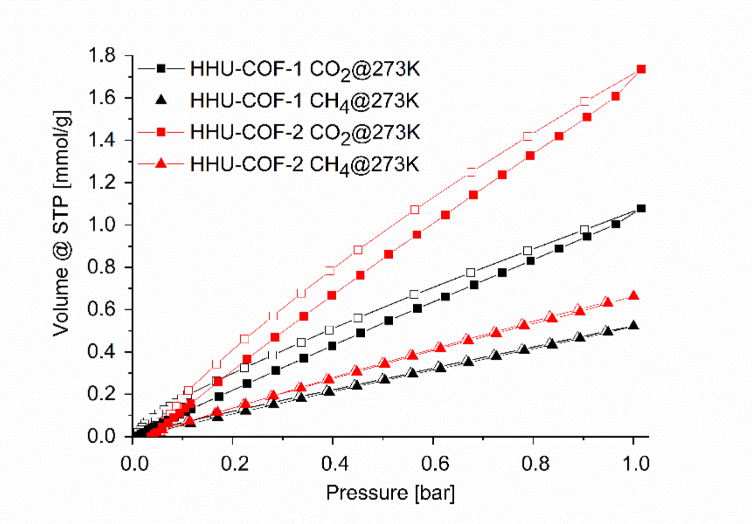

Figure S13. CO_2 and CH_4 sorption isotherms at 273 K of HHU-COF-1 and HHU-COF-2, respectively.

Table S3. Comparison of CO_2 uptake and BET surface area of imine-linked/azine COFs.

Material	CO_2 uptake, 1 bar, 273 K	BET surface area, N_2 at 77 K [m^2/g]	Reference
HHU-COF-1	1.08 mmol/g	2352	This work
HHU-COF-2	1.74 mmol/g	1356	
RT-COF-1[a]	44 cm^3/g (1.96 mmol/g)*	329	[2]
$[HO]_{25\%}$-H_2P-COF[b]	54 mg/g (1.23 mmol/g)	1054	[3]
$[HO]_{100\%}$-H_2P-COF[b]	63 mg/g (1.43 mmol/g)	1284	
$[HO_2C]_{50\%}$-H_2P-COF[c]	96 mg/g (2.18 mmol/g)	786	
$[HO_2C]_{100\%}$-H_2P-COF[c]	174 mg/g (3.95 mmol/g)	364	
DhaTph[d]	65 cm^3/g (2.90 mmol/g)	1305	[4]
DmaTph[e]	37 cm^3/g (1.65 mmol/g)	431	
TAPB-TFPB[f]	40.1 mg/g (0.91 mmol/g)	229	[5]
iPrTAPB-TFPB[g]	31.2 mg/g (0.71 mmol/g)	391	
TAPB-TFP[h]	180 mg/g (4.09 mmol/g)	567	
*i*PrTAPB-TFP[i]	105.2 mg/g (2.39 mmol/g)	756	
TpPa-1[j]	78 cm^3/g (3.48 mmol/g)	535	[6]
TpPa-2 [k]	64 cm^3/g (2.86 mmol/g)	339	

* data obtained from graph; [a] synthesized from 1,3,5-tris(4-aminophenyl)benzene (TAPB) and 1,3,5-benzenetricarbaldehyde (BTCA); [b] imine-linked 2D COF with porphyrin scaffold and phenol units on the pore walls; [c] based on $[HO]_{100\%}$-H_2P-COF with additional open carboxylic acid groups; [d] COF synthesized by reaction of 2,5-dihydroxyterephthalaldehyde (Dha) with 5,10,15,20-tetrakis(4-aminophenyl)-21*H*,23*H*-porphine (Tph); [e] COF synthesized by reaction of 2,5-dimethoxyterephthalaldehyde (Dma) with 5,10,15,20-tetrakis(4-aminophenyl)-21*H*,23*H*-porphine (Tph); [f] COF synthesized by reaction of 1,3,5-tris(4′-aminophenyl)benzene (TAPB) with 1,3,5-tris(4′-formylphenyl)benzene (TFPB); [g] COF synthesized by reaction of 1,3,5-tris(4′-amino-3′,5′-isopropylphenyl)benzene (*i*PrTAPB) with 1,3,5-tris(4′-formylphenyl)benzene (TFPB); [h] COF synthesized by reaction of 1,3,5-tris(4′-aminophenyl)benzene (TAPB) with 1,3,5-triformylphhluroglucinol (TFP); [i] COF synthesized by reaction of 1,3,5-tris(4′-amino-3′,5′-isopropylphenyl)benzene (*i*PrTAPB) with 1,3,5-triformylphhluroglucinol (TFP); [j] COF synthesized by reaction of 1,3,5-triformylphloroglucinol (Tp) with *p*-phenylenediamine (Pa-1); [k] COF synthesized by reaction of 1,3,5-triformylphloroglucinol (Tp) 2,5-dimethyl-*p*-phenylenediamine (Pa-2)

2.5. Ideal adsorbed solution theory (IAST) selectivities

The CO_2 and CH_4 isotherms of HHU-COF-1 were fitted with the Langmuir (LAI) isotherm model and the isotherms of HHU-COF-2 were fitted with the Toth model.

Table S4. Parameters for LAI and Toth fitting.

Gas	Temperature [K]	Model	R^2	Affinity constant *K* [1/bar]	Maximal loading q_{max} [mmol/g]	Heterogenity exponent t
HHU-COF-1						
CO_2	273	LAI	0.9999	0.070	15.870	-
CH_4	273	LAI	0.9999	0.393	16.221	-
HHU-COF-2						
CO_2	273	Toth	0.9962	0.501	3.343	7.762
CH_4	273	Toth	0.9996	0.440	1.551	2.273

The CO_2/CH_4 selectivities for a binary (50:50; v:v) mixture of the gases were calculated by applying the ideal adsorbed solution theory (IAST). **Figure S14** shows the IAST selectivities as a function of the pressure.

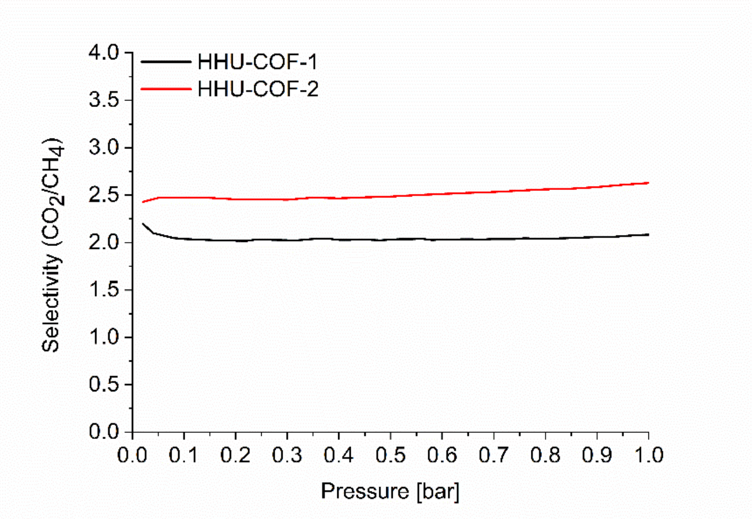

Figure S14. IAST selectivities of HHU-COF-1 and HHU-COF-2 for a binary (50:50; v:v) mixture of the gases CO_2/CH_4 at 273 K.

2.6. Thermogravimetric analysis (TGA)

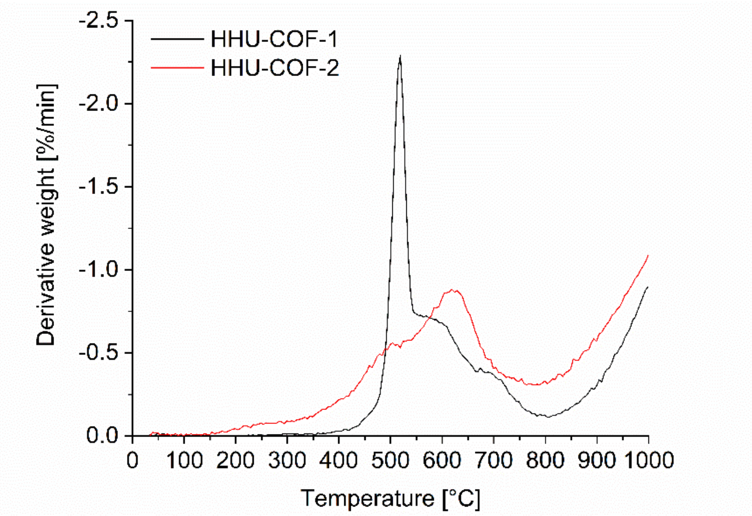

Figure S15. First derivative of TGA curves of HHU-COF-1 and HHU-COF-2. Measurement under nitrogen atmosphere with a heating rate of 5 K/min.

2.7. Correlation of 2theta values

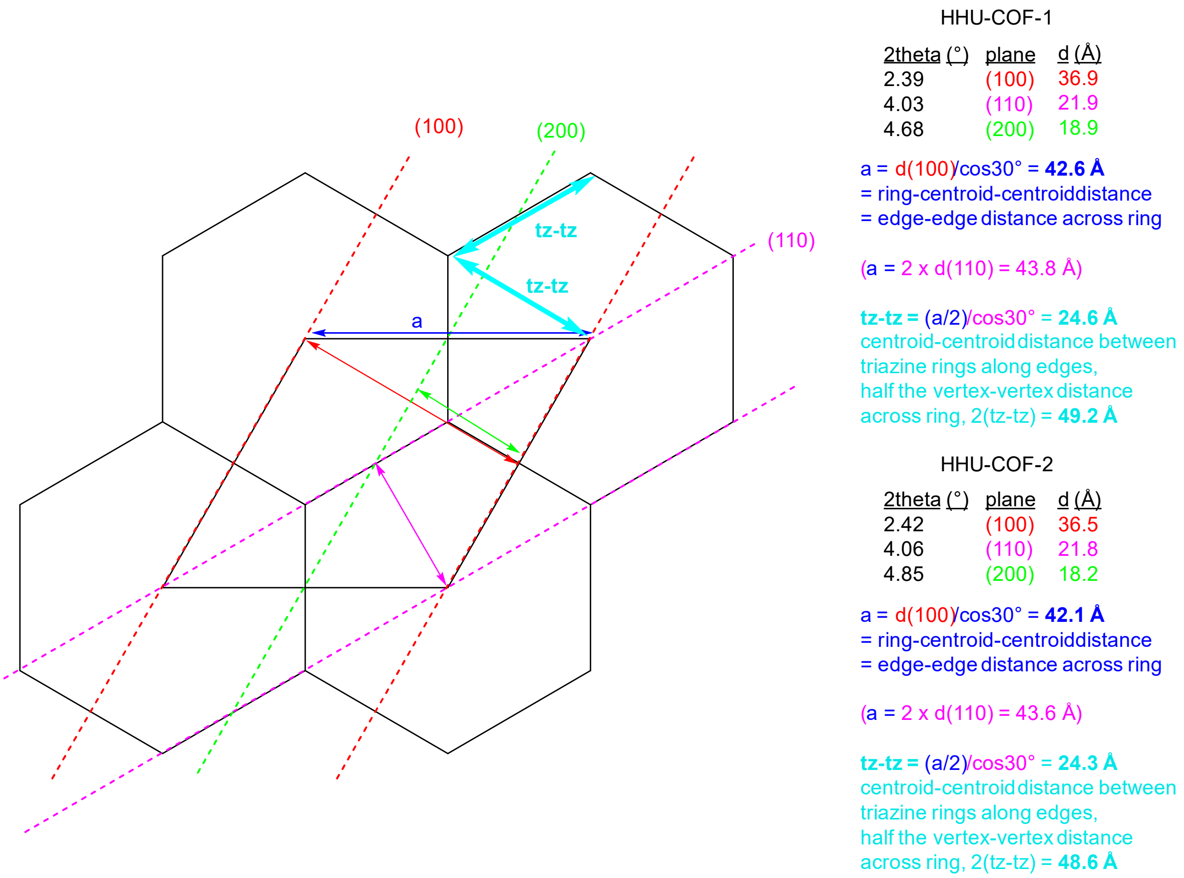

Figure S16. Correlation of the 2theta (2θ) values from the powder X-ray diffractograms in Figure 5 in the main text with the reflection planes and the d spacing according to the Bragg equation $n\lambda = 2d \sin\theta$ or $d = n\lambda/(2\sin\theta)$ with $\lambda = 1.5406$ Å and $n = 1$. Note that the edge-edge distances a and the triazine-centroid triazine-centroid (tz-tz) distances along the edge derived therefrom as (a/2)/cos30° were determined from the most intense and, thus, most accurately measurable (100) reflexes in the powder-X-ray diffractograms of HHU-COF-1 and -2.

2.8. Images of HHU-COF-1 and HHU-COF-2

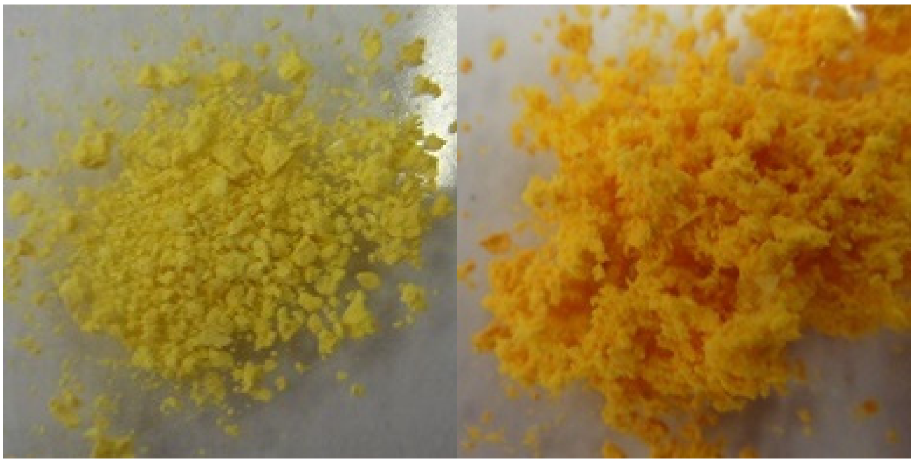

Figure S17. Images of HHU-COF-1 (left) and HHU-COF-2 (right).

3. Characterization of HHU-COF-1 (larger scale) and HHU-COF-2 (larger scale)

3.1. Infrared (IR) spectroscopy

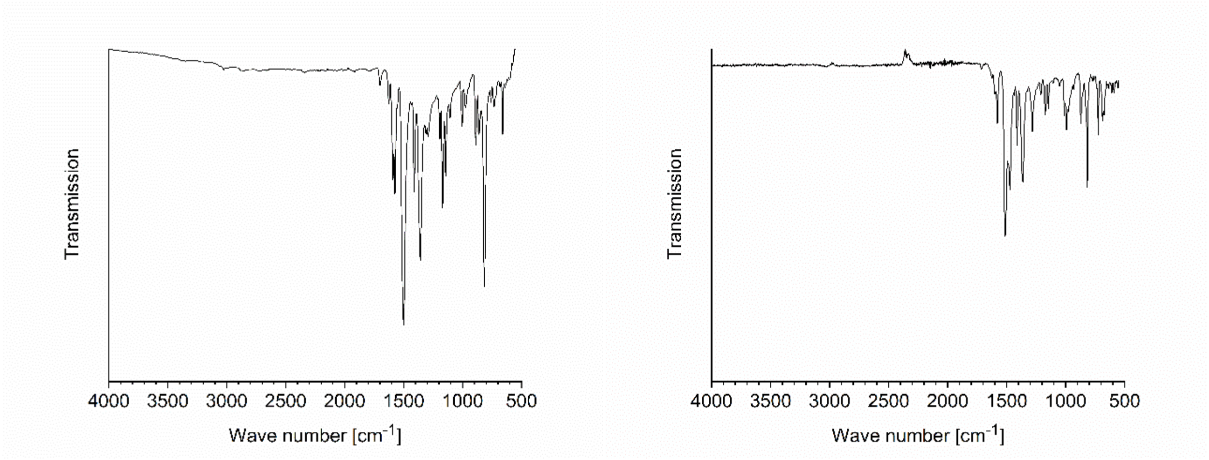

Figure S18. IR-spectra of HHU-COF-1 (larger scale; left) and HHU-COF-2 (larger scale; right).

3.2. Elemental analysis

Table S5. Elemental analysis of HHU-COF-1 (larger scale) and HHU-COF-2 (larger scale).

	C [wt%]	H [wt%]	N [wt%]	Rest [wt%]
HHU-COF-1 Calculated	81.93	4.42	13.65	-
HHU-COF-1 (larger scale)	80.86	4.27	13.03	1.84
HHU-COF-2 Calculated	60.66	1.82	10.11	27.41
HHU-COF-2 (larger scale)	60.23	1.75	10.01	28.01

3.3. N_2-sorption

The nitrogen sorption isotherm of HHU-COF-1 (larger scale) is shown in Figure S19. The BET surface area was determined as 2351 m^2/g and the total pore volume as 0.69 cm^3/g. The pore size distribution (Figure S19) showed a maximum at a pore diameter between 25 Å and 27 Å. Three further maxima were at pore diameters of 14 Å and 18 Å

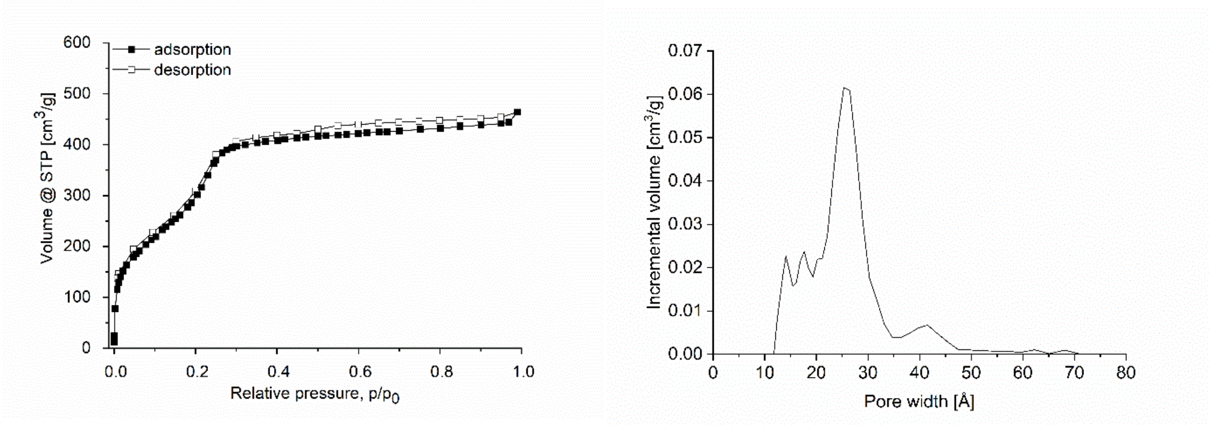

Figure S19. Nitrogen sorption isotherm (left) and pore size distribution calculated with slit pore, NLDFT equilibrium model (right) of HHU-COF-1 (larger scale).

The nitrogen sorption isotherm of HHU-COF-2 (larger scale) is shown in Figure S20. The BET surface area was determined as 1346 m^2/g and the total pore volume as 0.68 cm^3/g. The pore size distribution (Figure S20) mainly revealed pore diameters between 10 to 30 Å. In addition, a minor contribution of pores up to 200 Å in diameter was observed.

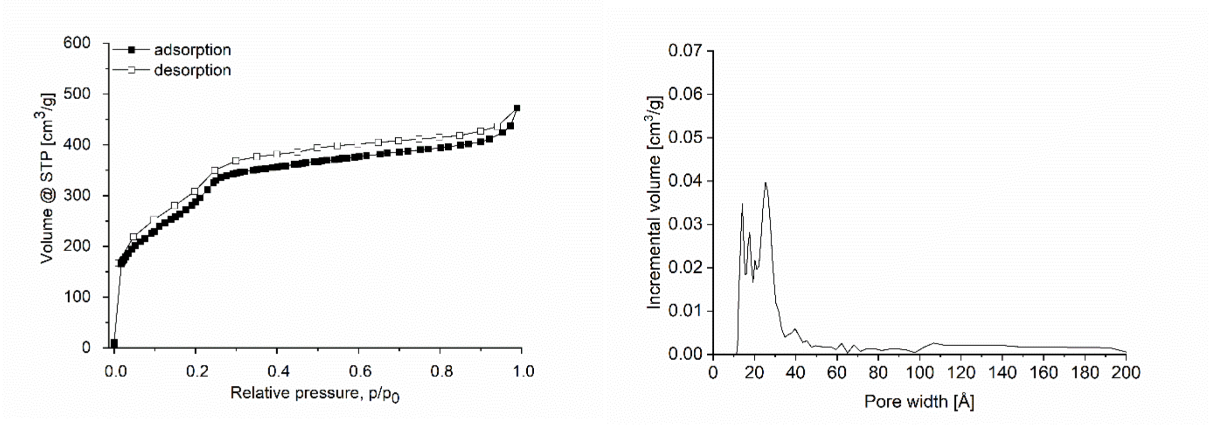

Figure S20. Nitrogen sorption isotherm (left) and pore size distribution calculated with slit pore, NLDFT equilibrium model (right) of HHU-COF-2 (larger scale).

3.4. TGA

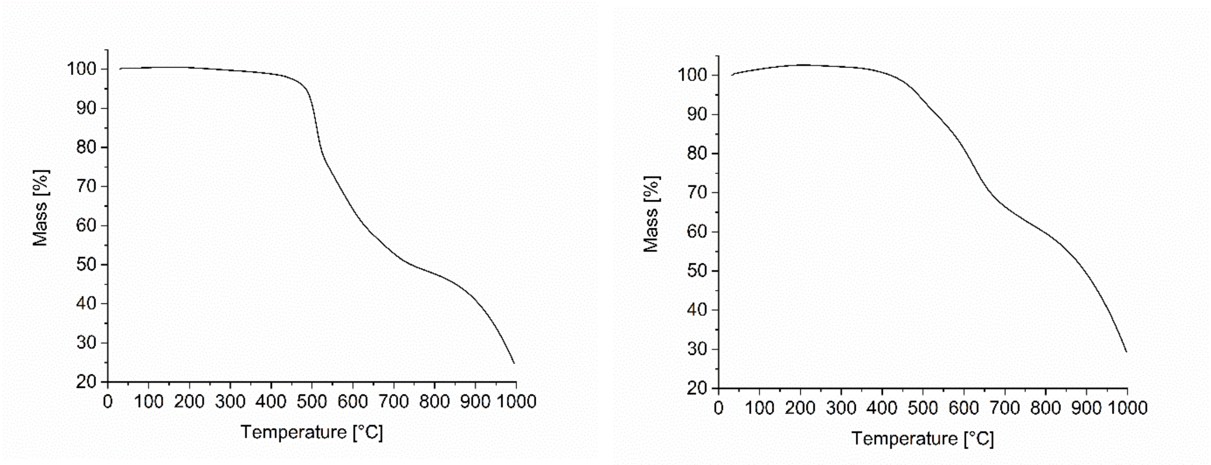

Figure S21. TGA curves of HHU-COF-1 (larger scale; left) and HHU-COF-2 (larger scale; right). Acquired under nitrogen atmosphere with a heating rate of 5 K/min.

3.5. Powder X-Ray diffraction (PXRD)

The PXRD of HHU-COF-1 (larger scale) (

Figure **S22**; left) showed, in addition to the characteristic reflex between 2° and 3° 2θ, two reflexes with lower intensity at 4° and about 5° 2θ. The PXRD of HHU-COF-2 (larger scale) (

Figure **S22**; right) exhibited a reflex between 2° and 3° 2θ. Another reflex could be observed at 5° 2θ. Both COFs showed no evidence of an amorphous character.

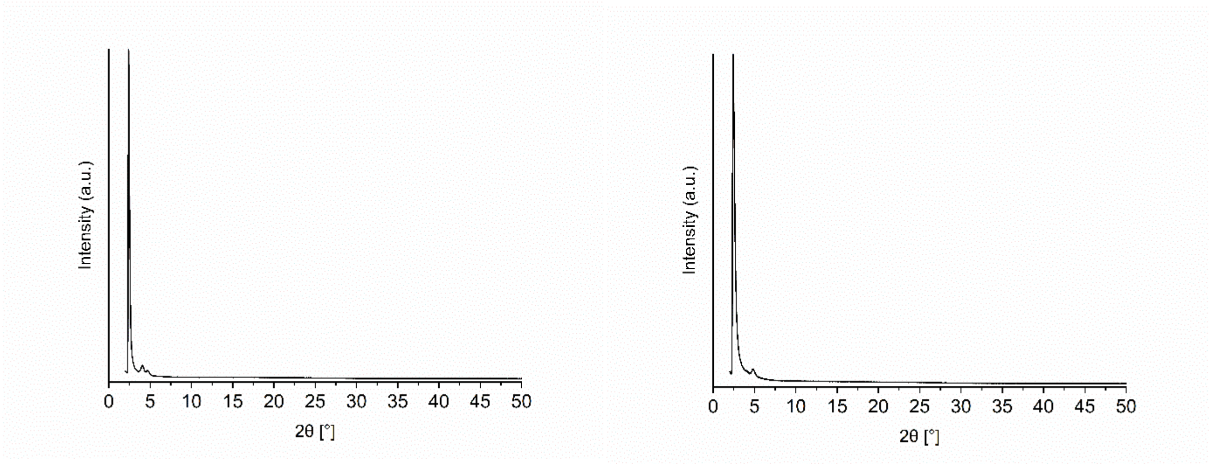

Figure S22. PXRD pattern of HHU-COF-1 (larger scale; left) and HHU-COF-2 (larger scale; right).

4. Preparation and characterization of MMMs

4.1. Schematic preparation of the pure polymer membrane and MMMs

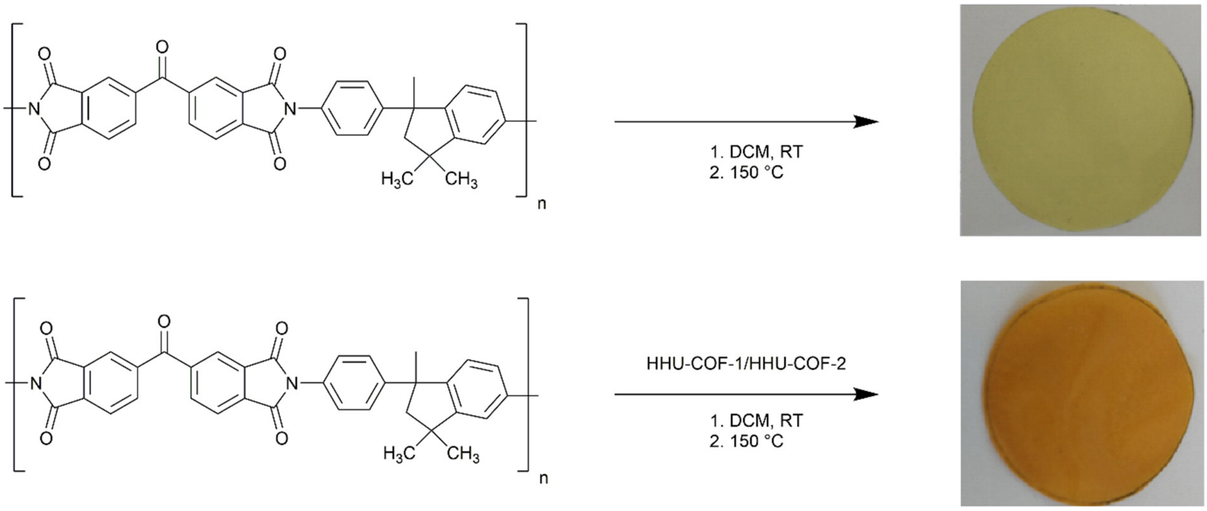

Figure S23. Schematic preparation of the pure Matrimid membrane (top) and MMMs, using the 16 wt% HHU-COF-2 MMM as an example (bottom).

4.2. Casting procedure

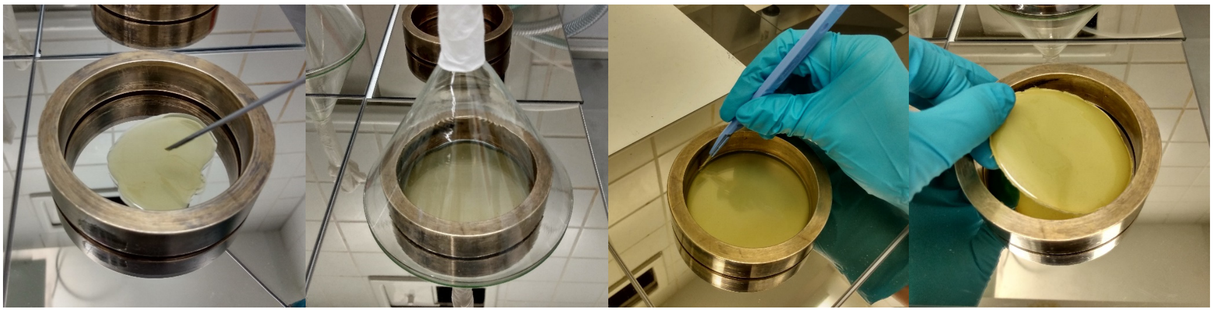

Figure S24. Preparation of membranes by solution casting (from left to right): casting the solution, drying, cutting with a scalpel and removing the membrane.

4.3. Set-up for CO_2/CH_4 mixed gas separation measurements

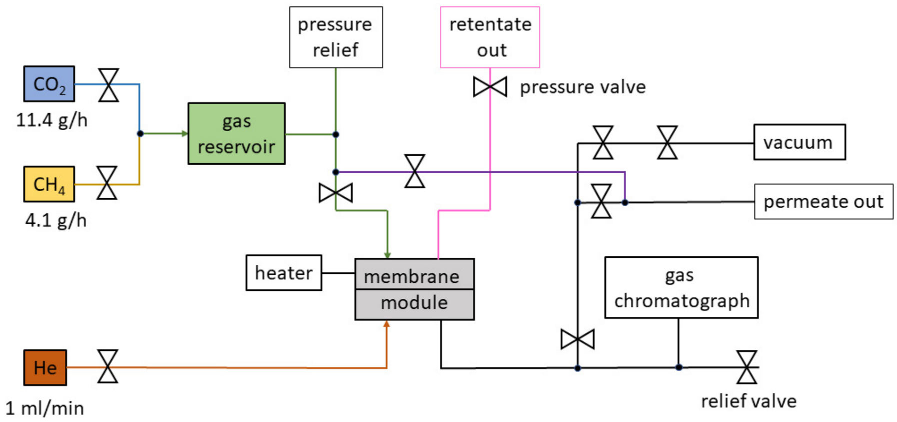

Figure S25. Set-up for CO_2/CH_4 mixed gas separation measurements [7].

4.4. Membrane thickness

Table S6. Average thickness of MMMs.

COF content [wt%]	HHU-COF-1/ Matrimid	HHU-COF-2/ Matrimid
	Average thickness [µm]	
8	74.9	74.6
16	72.4	68.6
24	73.0	73.0

4.5. SEM images of membrane surfaces

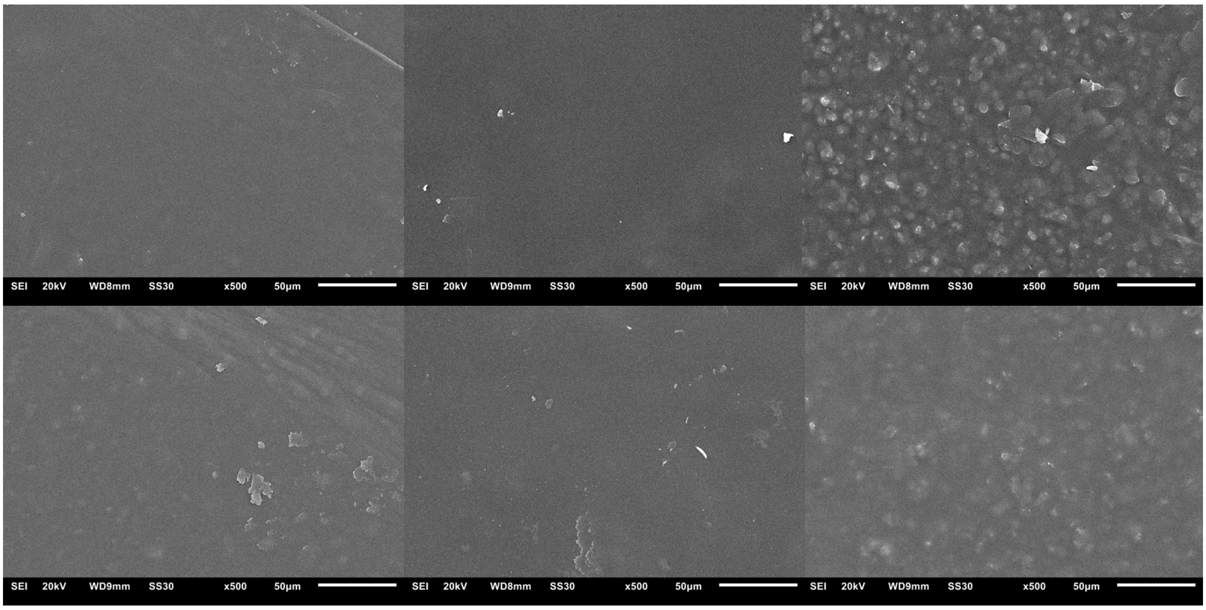

Figure S26. Top-surface SEM images of HHU-COF-1/Matrimid (top) with 8 wt% (left), 16 wt% (middle) and 24 wt% filler (right) and HHU-COF-2/Matrimid MMMs (bottom) with 8 wt% (left), 16 wt% (middle) and 24 wt% filler (right).

4.6. SEM-EDX of HHU-COF-2/Matrimid MMMs

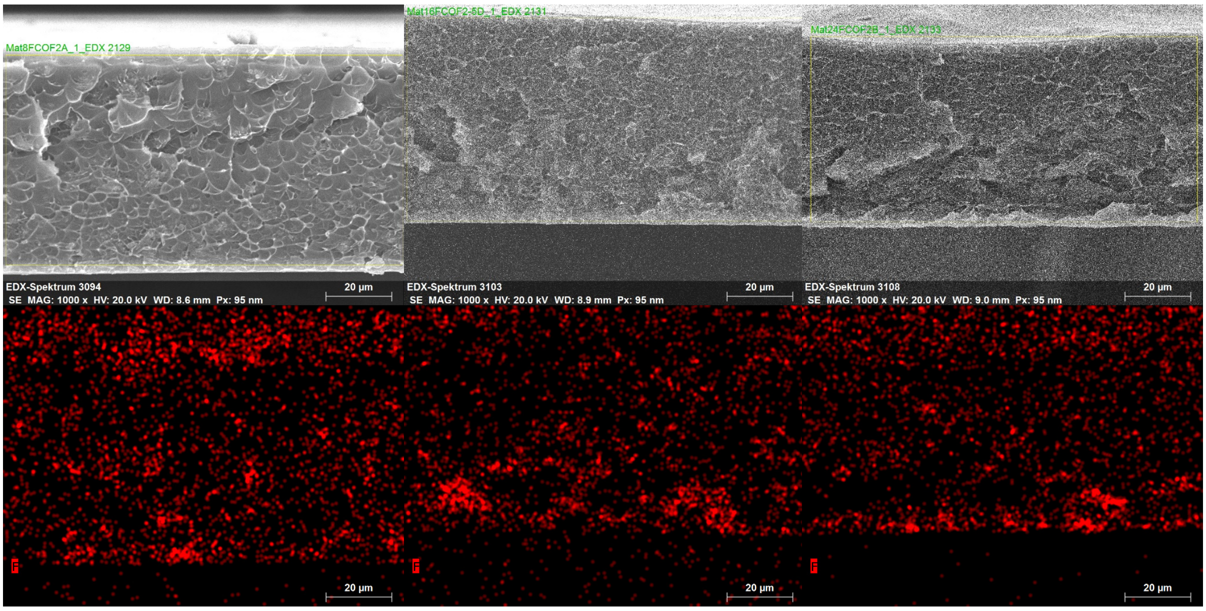

Figure S27. SEM images of HHU-COF-2/Matrimid MMMs (top) with 8 wt% (left), 16 wt% (middle) and 24 wt% filler (right) and associated fluorine elemental mapping (bottom).

4.7. Tensile strength

Table S7. Tensile strength of pure Matrimid and MMMs.

Filler material	-	HHU-COF-1			HHU-COF-2		
Filler content [wt%]	-	8	16	24	8	16	24
Tensile strength [MPa]	91	74	79	51	76	75	59

4.8. Long-term stability of MMMs

Table S8. Gas permeabilities (*P*) and mixed-gas selectivity factors (α) for COF/Matrimid MMMs when stored for one year under ambient conditions.

Filler material	Filler content [wt%]	P CO_2 [Barrer]	P CH_4 [Barrer]	α CO_2/CH_4
HHU-COF-1	8	8.6 ± 1.1	0.19 ± 0.03	44 ± 2
	16	8.0 ± 0.4	0.17 ± 0.01	46 ± 2
	24	5.8*	0.20*	28*
HHU-COF-2	8	7.1 ± 0.1	0.16 ± 0.01	45 ± 1
	16	10.5 ± 0.2	0.24 ± 0.01	44 ± 1
	24	12.9 ± 0.7	0.29 ± 0.02	45 ± 1

*Only one measurement due to breaking of the second MMM

4.9. Comparison of membrane performance

Table S9. Comparison of CO_2 permeability and CO_2/CH_4 selectivity for COFs/CTFs as porous filler materials in different polymer MMMs.

Filler	Filler content [wt%]	Matrix	*P* CO_2 [Barrer]	*P* CO_2 elevation [%]	*S/α* CO_2/CH_4	Ref.
-	-	Matrimid	6.8 ± 0.3	-	42 ± 1	This work
HHU-COF-1	8	Matrimid	9.1 ± 0.2	34	46 ± 2	
	16		9.1 ± 1.0	34	46 ± 1	
	24		5.8 ± 0.7	-	41 ± 1	
HHU-COF-2	8	Matrimid	7.1 ± 0.3	4	51 ± 1	
	16		10.2 ± 0.3	50	44 ± 2	
	24		13.0 ± 1.0	91	40 ± 1	
-	-	Matrimid	6.8 ± 0.1[a]	-	30.5± 0.6[a]	[8]
ACOF-1[1]	8	Matrimid	9.6 ± 1.0[a]	41	31.9 ± 0.8[a]	
	16		15.3 ± 0.7[a]	125	32.4 ± 1.8[a]	
COF-300	-	6FDA-DAM	767 ± 24[b]	-	22.3 ± 2.1[b]	[9]
	7	6FDA-DAM	1185 ± 41[b]	55	30.3 ± 1.5[b]	
	10		2842 ± 76[b]	271	24.6 ± 1.7[b]	
-	-	Pebax	73 ± 4[b]	-	18.7 ± 1.2[b]	
COF-300	10	Pebax	107 ± 6[b]	47	25.5 ± 1.3[b]	
-	-	Pebax	53[c]	-	17[c]	[10]
CTPP[2]	0.025	Pebax	73[c]	38	25[c]	
-	-	PIM-1[4]	3672[d]	-	10.6[d]	[11]
SNW-1[3]	10	PIM-1[4]	7553[d]	106	13.5[d]	
-	-	PIM-1[4]	5800[e]	-	11.5[e]	[12]
FCTF-1	2	PIM-1[4]	7300[e]	26	16.6[e]	
	5		9400[e]	62	14.8[e]	
-	-	Matrimid	6.8 ± 0.3[f]	-	42 ± 1[f]	[13]
CTF-biphenyl	8	Matrimid	12.0 ± 0.2[f]	76	43 ± 1[f]	
	16		15.1 ± 0.2[f]	122	44 ± 1[f]	
	24		15.4 ± 0.5[f]	126	44 ± 1[f]	
-	-	Matrimid	6.8 ± 0.3[f]	-	42 ± 1[f]	[7]
CTF-fluorene	8	Matrimid	9.2 ± 0.4[f]	35	43 ± 1[f]	
	16		12.6 ± 0.1[f]	85	45 ± 1[f]	
	24		17.8 ± 0.3[f]	162	44 ± 2[f]	

[a]Mixed gas; 308 K; feed pressure 4 bar; [b]Mixed gas; 298 K; transmembrane pressure 1 bar; [c]Single gas; 293 K; feed pressure 4 bar; [d]Single gas; 303 K; feed pressure 2 bar; [e]Single gas; 303 K; feed pressure 1 atm; [f]Mixed gas; 298 K; feed pressure 4 bar; [1]azine-linked covalent organic framework; [2]porous covalent triazine piperazine polymer; [3]Schiff base network; [4]polymer of intrinsic microporosity

5. Synthesis and characterization of TRITER-1 (= SCF-HCOF-1) and SCF-FCOF-1

5.1. Materials and Synthesis

Terephthalaldehyde (TA; 99%) was obtained from Sigma-Aldrich, 2,3,5,6-tetrafluoroterephthalaldehyde (TFTA; 98%) from BLDpharm and 1,3,5-tris-(4-aminophenyl)triazine (TAPT; > 98%) from TCI.

Synthesis of TRITER-1 (= SCF-HCOF-1)

TRITER-1 was synthesized in analogy to the literature [14]: 53.6 mg terephthalaldehyde (TA; 0.400 mmol), 94.4 mg TAPT (0.267 mmol) and 1 mL of the solvent mixture of 1,4-dioxane and mesitylene (1:1, v/v) were placed in a glass ampoule, followed by an ultra-sonification treatment for 15 min in order to ensure sufficient mixing of the educts. The mixture was degassed by applying three freeze-pump-thaw cycles and the ampoule was flame sealed under vacuum. After heating at 120 °C for three days, the crude product was washed with THF followed by Soxhlet extraction for 24 h each in THF and in ethanol to remove unreacted monomers. Drying was performed with supercritical CO_2. (yield: 113.0 mg; 84.4%).

Synthesis of SCF-FCOF-1

SCF-FCOF-1 was synthesized in analogy to the literature [14]: 82.4 mg 2,3,5,6-tetrafluoroterephthalaldehyde (TFTA; 0.400 mmol), 94.4 mg TAPT (0.267 mmol) and 1 mL of the solvent mixture of 1,4-dioxane and mesitylene (1:1, v/v) were placed in a glass ampoule, followed by an ultra-sonification treatment for 30 min in order to ensure sufficient mixing of the educts. The mixture was degassed by applying three freeze-pump-thaw cycles and the ampoule was flame sealed under vacuum. After heating at 120 °C for three days, the crude product was washed with THF followed by Soxhlet extraction for 24 h each in THF and in ethanol to remove unreacted monomers. Drying was performed with supercritical CO_2. (yield: 121.7 mg; 74.8%)

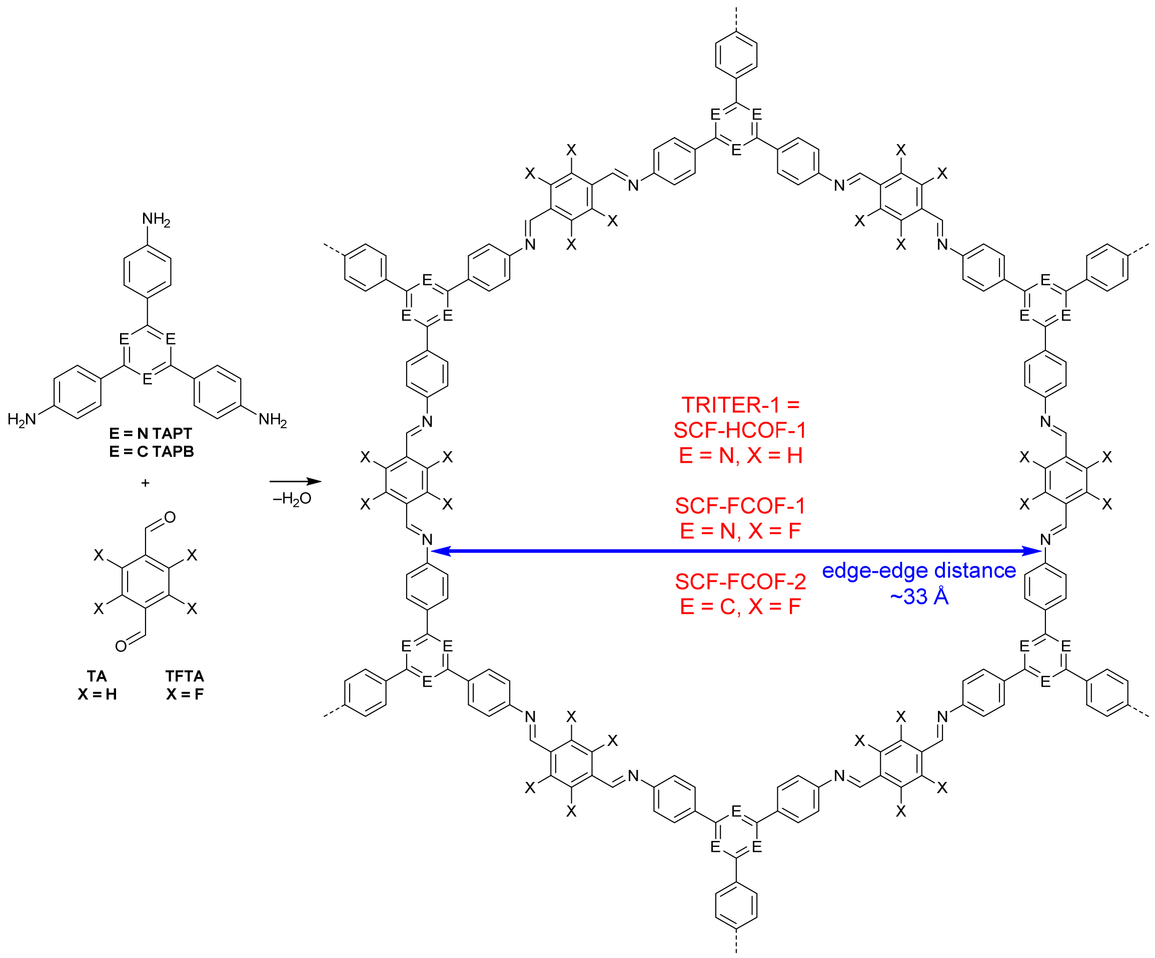

Scheme S1. Schematic formation of TRITER-1 (= SCF-HCOF-1) and SCF-FCOF-1 from TAPT and TA or TFTA, respectively [14,15]. (TAPB = 1,3,5-tris(4-aminophenyl) benzene, TAPT = 2,4,6-tris(4-aminophenyl)-1,3,5-triazine, TA = terephthalaldehyde, TFTA = 2,3,5,6-tetrafluoroterephthaldehyde). The edge-edge distance was taken from the literature of SCF-FCOF-1 and -2 [14].

5.2. IR spectroscopy

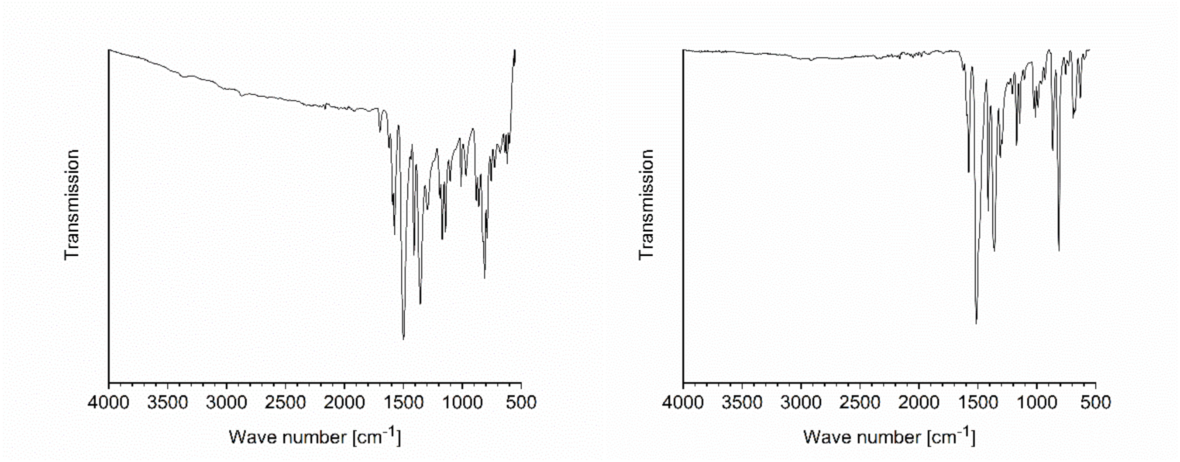

Figure S28. IR-spectra of TRITER-1 (left) and SCF-FCOF-1 (right).

5.3. Elemental analysis

Table S10. Elemental analysis of TRITER-1 and SCF-FCOF-1.

	C [wt%]	H [wt%]	N [wt%]	Rest [wt%]
TRITER-1 Calculated	79.04	4.19	16.77	-
TRITER-1	77.52	4.02	16.29	2.17
SCF-FCOF-1 Calculated	65.03	2.48	13.79	18.70
SCF-FCOF-1	65.19	2.67	13.64	18.50

5.4. SEM-EDX

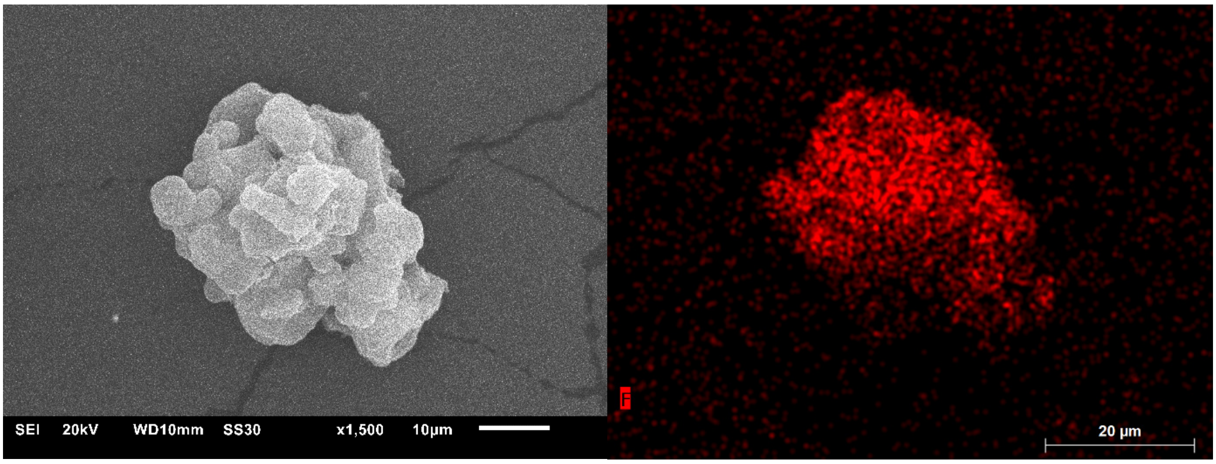

Figure S29. SEM image (left) of SCF-FCOF-1 and associated fluorine elemental mapping (right)

Table S11. EDX analysis of SCF-FCOF-1.

	C	H	F	N	O	Au	Si	Al	Sum
Ideal [wt%]	65.03	2.48	18.70	13.79	-	-	-	-	100
EDX [wt%]	47.9	-	9.5	6.7	0.9	8.6	0.2	0.1	73.9
EDX [wt%] without Au	47.9	-	9.5	6.7	0.9	-	0.2	0.1	65.3
EDX [wt%][a] without Au	73.4	-	14.5	10.3	1.4	-	0.3	0.2	100

[a] normalized

5.5. N_2-sorption

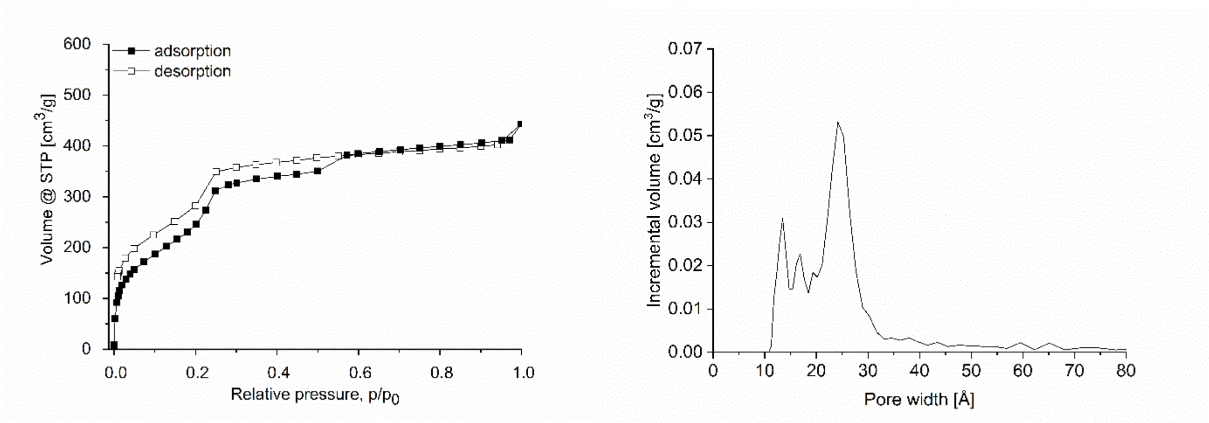

Figure S30. Nitrogen sorption isotherm (left) and pore size distribution calculated with slit pore, NLDFT equilibrium model (right) of TRITER-1.

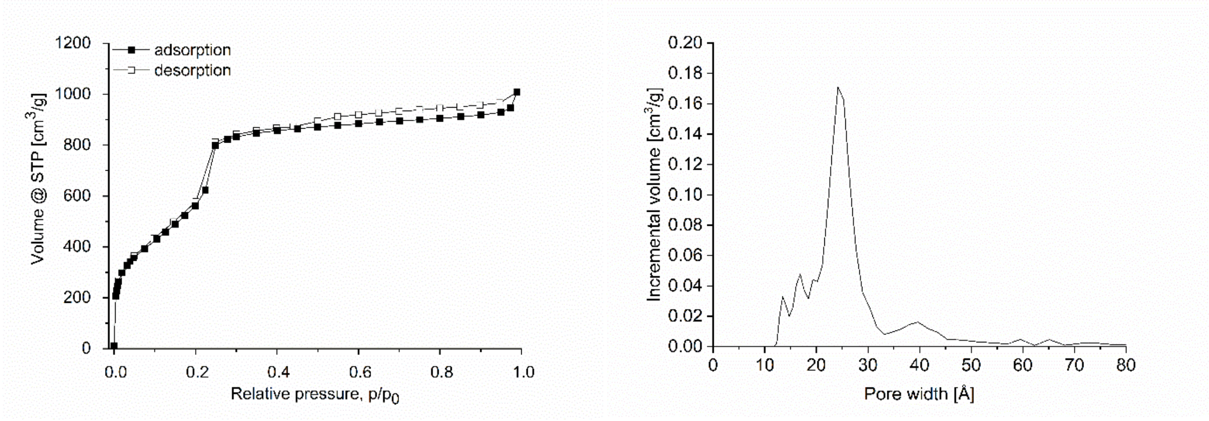

Figure S31. Nitrogen sorption isotherm (left) and pore size distribution calculated with slit pore, NLDFT equilibrium model (right) of SCF-FCOF-1.

5.6. TGA

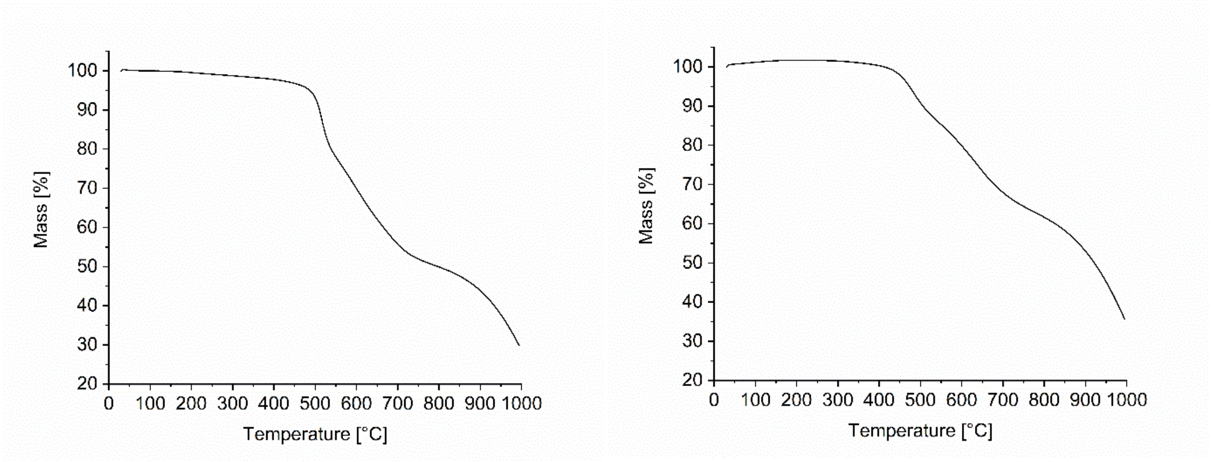

Figure S32. TGA curves of TRITER-1 (left) and SCF-FCOF-1 (right). Acquired under nitrogen atmosphere with a heating rate of 5 K/min.

5.7. PXRD

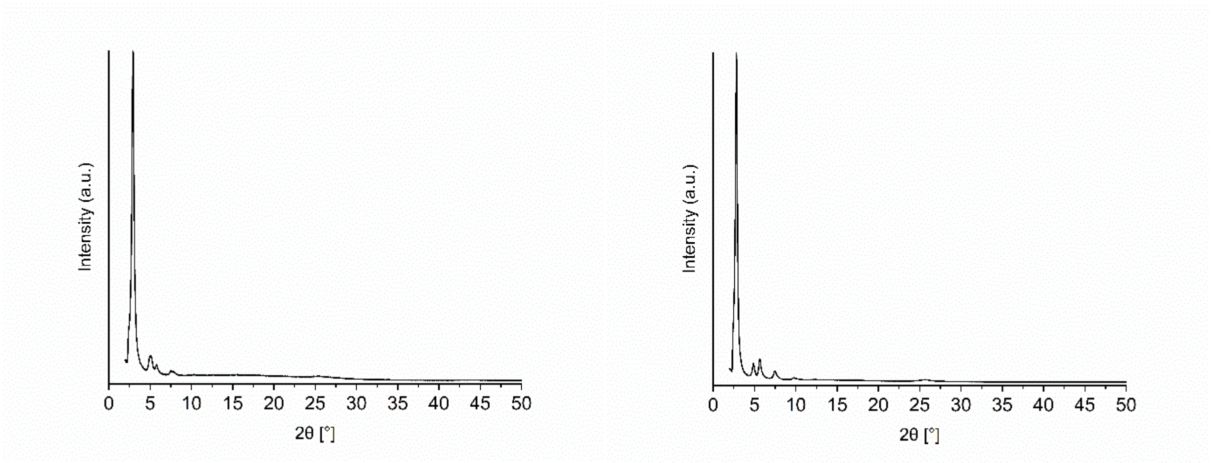

Figure S33. PXRD pattern of TRITER-1 (left) and SCF-FCOF-1 (right) prepared in this work.

5.8. Images of TRITER-1 and SCF-FCOF-1

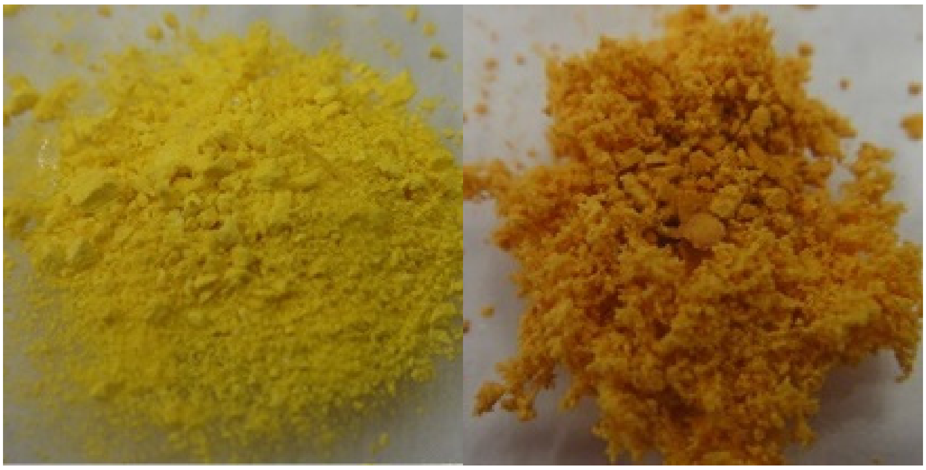

Figure S34. Images of TRITER-1 (left) and SCF-FCOF-1 (right).

6. References

1. Chen, T.-H.; Popov, I.; Zenasni, O.; Daugulis, O.; Miljanić, O. Š. Superhydrophobic perfluorinated metal–organic frameworks. *Chem. Commun.* **2013**, *49*, 6846–6848. DOI: 10.1039/C3CC41564C

2. de la Peña Ruigómez, A.; Rodríguez-San-Miguel, D.; Stylianou, K.C.; Cavallini, M.; Gentili, D.; Liscio, F.; Milita, S.; Roscioni , O.M.; Ruiz-González, M.L.; Carbonell, C.; Maspoch, D.; Mas-Ballesté, R.; Segura, J.L.; Zamora, F. Direct On-Surface Patterning of a Crystalline Laminar Covalent Organic Framework Synthesized at Room Temperature. *Chem. Eur. J.* **2015**, *21*, 10666–10670. DOI: 10.1002/chem.201501692

3. Huang, N.; Chen, X.; Krishna, R.; Jiang, D. Two-Dimensional Covalent Organic Frameworks for Carbon Dioxide Capture through Channel-Wall Functionalization. *Angew. Chem. Int. Ed.* **2015**, *54*, 2986–2990. DOI: 10.1002/anie.201411262

4. Kandambeth, S.; Shinde, D.B.; Panda, M.K.; Lukose, B.; Heine, T.; Banerjee, R. Enhancement of Chemical Stability and Crystallinity in Porphyrin-Containing Covalent Organic Frameworks by Intramolecular Hydrogen Bonds. *Angew. Chem. Int. Ed.* **2013**, *52*, 13052–13056. DOI: 10.1002/anie.201306775

5. Kaleeswaran, D.; Vishnoi, P.; Murugavel, R. [3+3] Imine and β-ketoenamine tethered fluorescent covalent-organic frameworks for CO_2 uptake and nitroaromatic sensing. *J. Mater. Chem. C* **2015**, *3*, 7159–7171. DOI: 10.1039/C5TC00670H

6. Kandambeth, S.; Mallick, A.; Lukose, B.; Mane, M.V.; Heine, T.; Banerjee, R. Construction of Crystalline 2D Covalent Organic Frameworks with Remarkable Chemical (Acid/Base) Stability via a Combined Reversible and Irreversible Route. *J. Am. Chem. Soc.* **2012**, *134*, 19524–19527. DOI: 10.1021/ja308278w

7. Bügel, S.; Spieß, A.; Janiak, C. Covalent triazine framework CTF-fluorene as porous filler material in mixed matrix membranes for CO_2/CH_4 separation. *Micropor. Mesopor. Mat.* **2021**, *316*, 110941. DOI: 10.1016/j.micromeso.2021.110941

8. Shan, M.; Seoane, B.; Rozhko, E.; Dikhtiarenko, A.; Clet, G.; Kapteijn, F.; Gascon, J. Azine-Linked Covalent Organic Framework (COF)-Based Mixed-Matrix Membranes for CO_2/CH_4 Separation. *Chem. Eur. J.* **2016**, *22*, 14467–14470. DOI: 10.1002/chem.201602999

9. Cheng, Y.; Zhai, L.; Ying, Y.; Wang, Y.; Liu, G.; Dong, J.; Ng, D.Z.L.; Khan, S.A.; Zhao, D. Highly efficient CO_2 capture by mixed matrix membranes containing three-dimensional covalent organic framework fillers. *J. Mater. Chem. A* **2019**, *7*, 4549–4560. DOI: 10.1039/C8TA10333J

10. Thankamony, R.L.; Li, X.; Das, S.K.; Ostwal, M.M.; Lai, Z. Porous covalent triazine piperazine polymer (CTPP)/PEBAX mixed matrix membranes for CO_2/N_2 and CO_2/CH_4 separations. *J. Membr. Sci.* **2019**, *591*, 117348. DOI: 10.1016/j.memsci.2019.117348

11. Wu, X.; Tian, Z.; Wang, S.; Peng, D.; Yang, L.; Wu, Y.; Xin, Q.; Wu, H.; Jiang, Z. Mixed matrix membranes comprising polymers of intrinsic microporosity and covalent organic framework for gas separation. *J. Membr. Sci.* **2017**, *528*, 273–283. DOI: 10.1016/j.memsci.2017.01.042

12. Jiang, H.; Zhang, J.; Huang, T.; Xue, J.; Ren, Y.; Guo, Z.; Wang, H.; Yang, L.; Yin, Y.; Jiang, Z.; Guiver, M.D. Mixed-Matrix Membranes with Covalent Triazine Framework Fillers in Polymers of Intrinsic Microporosity for CO_2 Separations. *Ind. Eng. Chem. Res.* **2019**, *59*, 5296–5306. DOI: 10.1021/acs.iecr.9b04632

13. Bügel, S.; Hoang, Q.-D.; Spieß, A.; Sun, Y.; Xing, S.; Janiak, C. Biphenyl-Based Covalent Triazine Framework/Matrimid® Mixed-Matrix Membranes for CO_2/CH_4 Separation. *Membranes* **2021**, *11*, 795. DOI: 10.3390/membranes11100795

14. Liao, Q.; Ke, C.; Huang, X.; Zhang, G.; Zhang, Q.; Zhang, Z.; Zhang, Y.; Liu, Y.; Ning, F.; Xi, K. Catalyst-free and efficient fabrication of highly crystalline fluorinated covalent organic frameworks for selective guest adsorption. *J. Mater. Chem. A* **2019**, *7*, 18959–18970. DOI: 10.1039/C9TA06214A

15. Gomes, R.; Bhanja, P.; Bhaumik, A. A triazine-based covalent organic polymer for efficient CO_2 adsorption. *Chem. Commun.* **2015**, *51*, 10050–10053. DOI: 10.1039/C5CC02147B
